# Phenotypic and Genotypic Characterization of Novel Polyvalent Bacteriophages With Potent *In Vitro* Activity Against an International Collection of Genetically Diverse *Staphylococcus aureus*

**DOI:** 10.3389/fcimb.2021.698909

**Published:** 2021-07-06

**Authors:** Elliot Whittard, James Redfern, Guoqing Xia, Andrew Millard, Roobinidevi Ragupathy, Sladjana Malic, Mark C. Enright

**Affiliations:** ^1^ Department of Life Sciences, Manchester Metropolitan University, Manchester, United Kingdom; ^2^ Lydia Becker Institute of Immunology and Inflammation, University of Manchester, Manchester, United Kingdom; ^3^ Department of Genetics and Genome Biology, University of Leicester, Leicester, United Kingdom

**Keywords:** *Staphylococcus aureus*, bacteriophage, biofilm, methicillin-resistant *Staphylococcus aureus*, genomics

## Abstract

Phage therapy recently passed a key milestone with success of the first regulated clinical trial using systemic administration. In this single-arm non-comparative safety study, phages were administered intravenously to patients with invasive *Staphylococcus aureus* infections with no adverse reactions reported. Here, we examined features of 78 lytic *S. aureus* phages, most of which were propagated using a *S. carnosus* host modified to be broadly susceptible to staphylococcal phage infection. Use of this host eliminates the threat of contamination with staphylococcal prophage — the main vector of *S. aureus* horizontal gene transfer. We determined the host range of these phages against an international collection of 185 *S. aureus* isolates with 56 different multilocus sequence types that included multiple representatives of all epidemic MRSA and MSSA clonal complexes. Forty of our 78 phages were able to infect > 90% of study isolates, 15 were able to infect > 95%, and two could infect all 184 clinical isolates, but not a phage-resistant mutant generated in a previous study. We selected the 10 phages with the widest host range for *in vitro* characterization by planktonic culture time-kill analysis against four isolates:- modified *S. carnosus* strain TM300H, methicillin-sensitive isolates D329 and 15981, and MRSA isolate 252. Six of these 10 phages were able to rapidly kill, reducing cell numbers of at least three isolates. The four best-performing phages, in this assay, were further shown to be highly effective in reducing 48 h biofilms on polystyrene formed by eight ST22 and eight ST36 MRSA isolates. Genomes of 22 of the widest host-range phages showed they belonged to the *Twortvirinae* subfamily of the order *Caudovirales* in three main groups corresponding to *Silviavirus*, and two distinct groups of *Kayvirus*. These genomes assembled as single-linear dsDNAs with an average length of 140 kb and a GC content of *c.* 30%. Phages that could infect > 96% of *S. aureus* isolates were found in all three groups, and these have great potential as therapeutic candidates if, in future studies, they can be formulated to maximize their efficacy and eliminate emergence of phage resistance by using appropriate combinations.****

## Introduction

*Staphylococcus aureus* is one of the major causes of both hospital- and community-acquired infections globally. It is an extremely versatile pathogen, causing a broad spectrum of diseases ranging in severity from minor skin and soft-tissue infections to life-threatening invasive infections (Lowy, 1998). Hospitalized patients are particularly prone to *S. aureus* infections because of the presence of compromised immune systems and surgical site infections caused by the implantation of indwelling medical devices (Brandt et al., 1999). *S. aureus* is commonly resistant to penicillin and methicillin-resistant *S. aureus* (MRSA) infections, resistant to all beta-lactam antibiotics, are most frequently treated with intravenous vancomycin, the antibiotic of “last resort” for resistant staphylococcal infections. However, resistance to this antibiotic (Centers for Disease Control and Prevention, 2002) and newer agent, such as linezolid (Tsiodras et al., 2001) and daptomycin (Skiest, 2006), have emerged, and there is a great and ever-increasing need for novel antibiotics and other therapeutic strategies for treating this pathogen.

Phage therapy exploits the natural ability of lytic bacteriophages (phages) to invade, multiply intracellularly, and then kill their host. Obligately, lytic phage are regarded as the most appropriate candidates in human health because they are capable of rapidly killing their host, greatly reducing the chances of bacteria developing phage resistance (Skurnik et al., 2007; Cui et al., 2017b). They also lack the required genetic factors for genome incorporation found in temperate phages. Despite widespread use in the 1930s, phage therapeutics were largely eclipsed by the discovery and clinical development of broad-spectrum antibiotics in western countries. However, phage therapy continues to be used at The G. Eliava Institute of Bacteriophage, Microbiology, and Virology in Tbilisi, Georgia and the Institute of Immunology and Experimental Therapy in Wroclaw, Poland, and these have become major centers for the development and application of phage therapy (Summers, 2001; Kutter et al., 2010). Success rates exceeding 85% have been reported in treating antibiotic-resistant infections caused by pathogens, including *S. aureus*, *Pseudomonas aeruginosa*, *Klebsiella pneumoniae*, and *Escherichia coli* in Poland (O’Flaherty et al., 2009) in studies involving large patient numbers. To date, there have been three early-stage clinical trials of phages for the treatment of *S. aureus* infections. In 2009, Rhoads et al. reported safety in a trial of phages against venous leg ulcers (Rhoads et al., 2009) and more recently McCallin et al. (2018) demonstrated the safety of a broad-spectrum phage cocktail in a placebo-controlled study, including nasal and oral administration. A recent study in Australia demonstrated safety of a good manufacturing practice — quality three-phage cocktail administered intravenously to 13 patients with invasive *S. aureus* infections (Petrovic Fabijan et al., 2020). This study marked a milestone in phage therapy, the first regulated clinical trial of systemically administered phages.

Most reports in the literature regarding lytic staphylococcal phages show that these are typically members of the *Twortvirinae* subfamily, which currently comprises five genera representing distinct lineages — *Kayvirus*, *Sepunavirus*, *Silviavirus*, *Twortvirus*, and unclassified *Twortvirinae*. These phages display a broad host range, infecting most *S. aureus* isolates and even those of other staphylococcal species (Lobocka et al., 2012). Studies on these group of phages and also their lytic enzymes hold promise for future clinical development alone or in combination with antibiotics (Berryhill et al., 2021) especially as comparative genomic analysis show little similarity between phages from different lineages in gene complement or in their lytic gene sequences (O’Flaherty et al., 2005a; Synnott et al., 2009; Lobocka et al., 2012). These may therefore represent an important and diverse source of phages for traditional therapy or development of treatments based on novel antimicrobial enzymes.

Here, we report on the isolation and characterization of 78 lytic phages with broad host range against a diverse collection of *S. aureus.* These 185 isolates include members of all globally disseminated MRSA and methicillin-sensitive lineages, including multiple isolates of clonal complexes (CCs) 1, 5, 8, 22, 30, 45, 59, and CC80 containing the main MRSA lineages (Chatterjee and Otto, 2013). Wherever possible, we isolated and propagated phages on a modified *S. carnosus* isolate that is avirulent (Rosenstein et al., 2009), containing none of the virulence genes associated with *S. aureus* prophages that could potentially compromise the safe production of phages for therapeutic use. We examined the *in vitro* characteristics of selected phages in planktonic and biofilm culture and characterized the genomic similarity and taxonomy of 22 phages with some of the broadest host ranges.

## Materials and Methods

### Bacterial Strains

A modified *S. carnosus* strain, TM300H, a hybrid strain derived from TM300H expressing both its native glycerol-phosphate (GroP)- and *S. aureus* ribitol-phosphate (RboP)-type wall teichoic acids, was used for phage isolation and propagation. *S. carnosus* is a non-virulent staphylococcal species used in meat production, and as such, we considered it a benign propagating host for phage production. TM300H was transformed with a chloramphenicol resistance plasmid encoding polyribitol-phosphate (RboP) repeating units of *S. aureus* wall teichoic acid to promote phage adsorption (Winstel et al., 2013). This required supplementation of growth medium with 10 μg/ml chloramphenicol. The methicillin-sensitive isolate D329 was used as an alternative host for phage propagation where this was not possible using TM300H. *S. aureus* isolate 15981 (obtained from Prof ATA Jenkins, University of Bath, UK) was also included in this study as it is a very strong biofilm producer and has been used in several studies of *S. aureus* virulence and biofilm regulation including those by Valles et al. (2003) and Toledo-Arana et al. (2005). All bacterial isolates were cultured in tryptone soy broth (TSB) or agar (TSA) and stored at −80°C in TSB containing 25% (v/v) glycerol. One hundred eighty-five genetically diverse isolates of *S. aureus* from our collection, including isolates from 13 published studies of human and animal carriage and disease, were used in this study (**Table 1**). They comprised 126 MRSA and 59 MSSA isolates from 14 different countries with 58 different multilocus sequence types (Enright et al., 2000). This collection contains multiple representative isolates of all major MRSA and MSSA lineages (Enright et al., 2002; Feil et al., 2003; Holden et al., 2013), including those associated with community-onset (Vandenesch et al., 2003) and livestock-associated (van Belkum et al., 2008) MRSA infections. **Table 1** shows the MLST sequence type and clonal complex of each isolate, as well as a reference to the original study where the isolate was first characterized using MLST, and for MRSA isolates — SCC*mec* typing. Further information on study isolates are available from the cited source and, in most cases, from the PubMLST website at https://pubmlst.org/organisms/staphylococcus-aureus.

**Table 1:** Details of *Staphylococcus aureus* isolates used in this study.

Isolate	ST	CC	MRSA	Country	Year	Reference	Isolate	ST	CC	MRSA	Country	Year	Reference	Isolate	ST	CC	MRSA	Country	Year	Reference
H462	1	1	N	UK	1997	(Avrani and Lindell, 2015)	ARI7	22	22	Y	UK	2007	(Berryhill et al., 2021)	D97	55		N	UK	1997	(Beeton et al., 2015)
NL0118512	1	1	N	Netherlands	1999	(Bankevich et al., 2012)	F86956	22	22	Y	UK	2007	(Berryhill et al., 2021)	D318	57	30	N	UK	1997	(Beeton et al., 2015)
BTN2164	1	1	N	UK	1999	(Cui et al., 2017a)	H43162	22	22	Y	UK	2008	(Berryhill et al., 2021)	D508	58	15	N	UK	1997	(Beeton et al., 2015)
HT2001-254	1	1	Y	USA	2001	(Dickey and Perrot, 2019)	H91491	22	22	Y	UK	2007	(Berryhill et al., 2021)	D535	59		N	UK	1997	(Beeton et al., 2015)
A93-0066	5	5	Y	France	1993	(Adriaenssens and Brister, 2017)	HO50960412	22	22	Y	UK	2005	(Berryhill et al., 2021)	D551	59		N	UK	1997	(Beeton et al., 2015)
FIN61974	5	5	Y	Finland	2002	(Adriaenssens and Brister, 2017)	HO5322054809	22	22	Y	UK	2005	(Berryhill et al., 2021)	D473	69	1	N	UK	1997	(Beeton et al., 2015)
H157	5	5	N	UK	1997	(Avrani and Lindell, 2015)	HO72300407/05	22	22	Y	UK	2007	(Berryhill et al., 2021)	CDCUSA700	72	8	Y	USA	1998	(Chatterjee and Otto, 2013)
AR110735	5	5	Y	Ireland	1993	(Bankevich et al., 2012)	HO73740468/05	22	22	Y	UK	2007	(Berryhill et al., 2021)	SWEDEN8890/99	80	80	Y	Sweden	1999	(Adriaenssens and Brister, 2017)
BK519	5	5	Y	USA	1991	(Bankevich et al., 2012)	M81008	22	22	Y	UK	2007	(Berryhill et al., 2021)	HT2002-0664	80	80	Y	France	2002	(Dickey and Perrot, 2019)
NJ992	5	5	Y	USA	2002	(Bankevich et al., 2012)	T27706	22	22	Y	UK	2008	(Berryhill et al., 2021)	HT20040991	80	80	Y	France	2004	(Dickey and Perrot, 2019)
D10	5	5	N	UK	1997	(Beeton et al., 2015)	T50530	22	22	Y	UK	2007	(Berryhill et al., 2021)	BK1563	88		Y	USA	1991	(Bankevich et al., 2012)
CDC-USA800	5	5	Y	USA	1998	(Chatterjee and Otto, 2013)	W44936	22	22	Y	UK	2008	(Berryhill et al., 2021)	HT2001-0634	93	93	Y	Australia	2001	(Dickey and Perrot, 2019)
BTN2242	5	5	N	UK	2002	(Cui et al., 2017a)	370.07	22	22	Y	UK	2007	(Berryhill et al., 2021)	HT2002-0635	93	93	Y	Australia	2002	(Dickey and Perrot, 2019)
C56	6	5	N	UK	1997	(Avrani and Lindell, 2015)	99ST18131	22	22	Y	Australia	1999	(Berryhill et al., 2021)	H560	121		N	UK	1998	(Avrani and Lindell, 2015)
E228	8	8	N	Denmark	1957	(Alves et al., 2014)	RH06000061/09	22	22	N	UK	2000	(Brandt et al., 1999)	D139	145		N	UK	1997	(Beeton et al., 2015)
99ST22111	8	8	Y	Australia	1997	(Bankevich et al., 2012)	BTN1626	22	22	Y	UK	2002	(Cui et al., 2017a)	FIN62305	156		Y	Finland	1990	(Adriaenssens and Brister, 2017)
EMRSA13	8	8	Y	UK	1999	(Bankevich et al., 2012)	C49	23	22	N	UK	1997	(Avrani and Lindell, 2015)	D22	182		N	UK	1997	(Beeton et al., 2015)
EMRSA2	8	8	Y	UK	1999	(Bankevich et al., 2012)	D279	25		N	UK	1997	(Beeton et al., 2015)	CAN6428-011	188	1	N	Canada	2002	(Bankevich et al., 2012)
EMRSA6	8	8	Y	UK	1999	(Bankevich et al., 2012)	H118	28		N	UK	1997	(Avrani and Lindell, 2015)	D470	207		N	UK	1997	(Beeton et al., 2015)
EMRSA7	8	8	Y	UK	1999	(Bankevich et al., 2012)	CUBA4030	30	30	N	Cuba	2000	(Bankevich et al., 2012)	NOT116	227		N	UK	2000	(Brandt et al., 1999)
NL010548-1	8	8	N	Netherlands	1999	(Bankevich et al., 2012)	C390	31	30	N	UK	1997	(Avrani and Lindell, 2015)	WW2594/97-2	228	5	Y	Germany	1997	(Adriaenssens and Brister, 2017)
D137	8	8	N	UK	1997	(Beeton et al., 2015)	H399	33	30	N	UK	1997	(Avrani and Lindell, 2015)	GERMANY131/98	228	5	Y	Germany	1998	(Bankevich et al., 2012)
NOT110	8	8	N	UK	2000	(Brandt et al., 1999)	C160	34	30	N	UK	1997	(Avrani and Lindell, 2015)	CDC16	231	5	Y	USA	1998	(Chatterjee and Otto, 2013)
CDC-USA300	8	8	Y	USA	1998	(Chatterjee and Otto, 2013)	MRSA252ΦKmut	36	30	Y	UK	2013	(Abedon, 1992)	99.3759.V	235	5	Y	UK	2002	(Bankevich et al., 2012)
15981	8	8	N	Spain	2003	(Cui et al., 2017b)	FIN75916	36	30	Y	Finland	1996	(Adriaenssens and Brister, 2017)	91-4990	239	8	Y	Netherlands	1991	(Bankevich et al., 2012)
H169	9	1	N	UK	1997	(Avrani and Lindell, 2015)	UK96/32010	36	30	Y	UK	1996	(Adriaenssens and Brister, 2017)	EMRSA11	239	8	Y	UK	1999	(Bankevich et al., 2012)
D295	9	1	N	UK	1997	(Beeton et al., 2015)	H119MRSA	36	30	Y	UK	1997	(Avrani and Lindell, 2015)	EMRSA4	239	8	Y	UK	1999	(Bankevich et al., 2012)
D316	11	12	N	UK	1997	(Beeton et al., 2015)	H325	36	30	N	UK	1997	(Avrani and Lindell, 2015)	FFP200	239	8	Y	Portugal	1996	(Bankevich et al., 2012)
H117	12	12	N	UK	1997	(Avrani and Lindell, 2015)	MRSA252	36	30	Y	UK	1997	(Avrani and Lindell, 2015)	EMRSA9	240	8	Y	UK	1999	(Bankevich et al., 2012)
D329	12	12	N	UK	1997	(Beeton et al., 2015)	EMRSA16	36	30	Y	UK	1999	(Bankevich et al., 2012)	SWEDEN408/99	246	8	Y	Sweden	1999	(Adriaenssens and Brister, 2017)
H402	13		N	UK	1997	(Avrani and Lindell, 2015)	NottmA	36	30	Y	UK	2000	(Brandt et al., 1999)	FRA97393	247	8	Y	France	2002	(Adriaenssens and Brister, 2017)
C154	14	15	N	UK	1997	(Avrani and Lindell, 2015)	NottmA2	36	30	N	UK	2000	(Brandt et al., 1999)	82MRSA	247	8	Y	UK	1997	(Avrani and Lindell, 2015)
C357	15	15	N	UK	1997	(Avrani and Lindell, 2015)	03.1791.F	36	30	Y	UK	2003	(Centers for Disease Control and Prevention, 2002)	EMRSA5	247	8	Y	UK	1999	(Bankevich et al., 2012)
H291	18	15	N	UK	1997	(Avrani and Lindell, 2015)	06.9570.L	36	30	Y	UK	2006	(Centers for Disease Control and Prevention, 2002)	EMRSA8	250	8	Y	UK	1999	(Bankevich et al., 2012)
D17	20		N	UK	1997	(Beeton et al., 2015)	07.1227.Z	36	30	Y	UK	2007	(Centers for Disease Control and Prevention, 2002)	KD12168	250	8	Y	UK	1965	(Bankevich et al., 2012)
98/10618	22	22	Y	UK	1998	(Adriaenssens and Brister, 2017)	07.1696.F	36	30	Y	UK	2007	(Centers for Disease Control and Prevention, 2002)	27969	398	398	Y	UK	2012	N/A
SwedenAO9973	22	22	Y	Sweden	1999	(Adriaenssens and Brister, 2017)	07.2449.K	36	30	Y	UK	2007	(Centers for Disease Control and Prevention, 2002)	09.4620.V	398	398	Y	UK	2012	N/A
WW1678/96	22	22	Y	Germany	1996	(Adriaenssens and Brister, 2017)	07.2496.L	36	30	Y	UK	2007	(Centers for Disease Control and Prevention, 2002)	09.6440.M	398	398	Y	UK	2012	N/A
C101	22	22	N	UK	1997	(Avrani and Lindell, 2015)	07.2589.M	36	30	Y	UK	2007	(Centers for Disease Control and Prevention, 2002)	11.1299.J	398	398	Y	UK	2012	N/A
C720	22	22	Y	UK	1998	(Avrani and Lindell, 2015)	07.2880.V	36	30	Y	UK	2007	(Centers for Disease Control and Prevention, 2002)	11.2530.K	398	398	Y	UK	2012	N/A
H182MRSA	22	22	Y	UK	1997	(Avrani and Lindell, 2015)	07.3841.N	36	30	Y	UK	2007	(Centers for Disease Control and Prevention, 2002)	11.3281.H	398	398	Y	UK	2012	N/A
H65	22	22	N	UK	1998	(Avrani and Lindell, 2015)	07.6636.Y	36	30	Y	UK	2007	(Centers for Disease Control and Prevention, 2002)	11.4910.K	398	398	Y	UK	2012	N/A
EMRSA15-90	22	22	Y	UK	1990	(Bankevich et al., 2012)	07.6659.K	36	30	Y	UK	2007	(Centers for Disease Control and Prevention, 2002)	11.5252.H	398	398	Y	UK	2012	N/A
NL011399-5	22	22	N	Netherlands	1999	(Bankevich et al., 2012)	07.7206.Y	36	30	Y	UK	2007	(Centers for Disease Control and Prevention, 2002)	11.5654.T	398	398	Y	UK	2012	N/A
403.02	22	22	Y	UK	2002	(Berryhill et al., 2021)	97.2483.Hb	36	30	Y	UK	1997	(Centers for Disease Control and Prevention, 2002)	12.2167.C	398	398	Y	UK	2012	N/A
434.07	22	22	Y	UK	2007	(Berryhill et al., 2021)	98.5806.F	36	30	Y	UK	1998	(Centers for Disease Control and Prevention, 2002)	12.2539.L	398	398	Y	UK	2012	N/A
723.07	22	22	N	UK	2007	(Berryhill et al., 2021)	USA200	36	30	Y	USA	1998	(Chatterjee and Otto, 2013)	12.2732.H	398	398	Y	UK	2012	N/A
921.07	22	22	Y	UK	2007	(Berryhill et al., 2021)	BTN1429	36	30	Y	UK	2002	(Cui et al., 2017a)	42-57	398	398	Y	UK	2012	N/A
930.02	22	22	Y	UK	2002	(Berryhill et al., 2021)	BTN2172	36	30	Y	UK	2002	(Cui et al., 2017a)	BVCA92	398	398	Y	UK	2012	N/A
1018.07	22	22	Y	UK	2007	(Berryhill et al., 2021)	BTN2292	36	30	Y	UK	2002	(Cui et al., 2017a)	C7-t011	398	398	Y	UK	2007	N/A
1091	22	22	Y	UK	2008	(Berryhill et al., 2021)	BTN766	36	30	Y	UK	2002	(Cui et al., 2017a)	C7(P11)	398	398	Y	UK	2007	N/A
729192	22	22	Y	UK	2007	(Berryhill et al., 2021)	H137	38	30	N	UK	1997	(Avrani and Lindell, 2015)	C7(P4)	398	398	Y	UK	2007	N/A
98.4823.X	22	22	Y	UK	1998	(Berryhill et al., 2021)	C253	40	30	N	UK	1997	(Avrani and Lindell, 2015)	GKP136-53	398	398	Y	UK	2012	N/A
AR0650784	22	22	Y	Ireland	1993	(Berryhill et al., 2021)	C427	42		N	UK	1997	(Avrani and Lindell, 2015)	h-RVC57276	398	398	Y	UK	2012	N/A
ARI10	22	22	Y	UK	2007	(Berryhill et al., 2021)	FIN76167	45	45	Y	Finland	1996	(Adriaenssens and Brister, 2017)	m-38-53	398	398	Y	UK	2012	N/A
ARI11	22	22	Y	UK	2007	(Berryhill et al., 2021)	BTN2299	45	45	Y	UK	1999	(Cui et al., 2017a)	m-mecA-17-57	398	398	Y	UK	2012	N/A
ARI12	22	22	Y	UK	2007	(Berryhill et al., 2021)	BTN2306	45	45	Y	UK	1999	(Cui et al., 2017a)	RV2007-06745-3’A’	398	398	Y	UK	2007	N/A
ARI15	22	22	Y	UK	2007	(Berryhill et al., 2021)	C316	49		N	UK	1997	(Avrani and Lindell, 2015)	RV2007-13689-13	398	398	Y	UK	2007	N/A
ARI26	22	22	Y	UK	2007	(Berryhill et al., 2021)	H417	50		N	UK	1997	(Avrani and Lindell, 2015)	NOT161	843	97	N	UK	2000	(Brandt et al., 1999)
ARI31	22	22	Y	UK	2007	(Berryhill et al., 2021)	C3	51		N	UK	1997	(Avrani and Lindell, 2015)	NOT290	848	1	N	UK	2000	(Brandt et al., 1999)
ARI4	22	22	Y	UK	2007	(Berryhill et al., 2021)	D49	53	45	N	UK	1997	(Beeton et al., 2015)	BTN2289	868	5	N	UK	1999	(Cui et al., 2017a)
ARI5	22	22	Y	UK	2007	(Berryhill et al., 2021)	D98	54	45	N	UK	1997	(Beeton et al., 2015)							

### Phage Isolation, Propagation, and Host Range Determination

Sewage effluent samples were collected from various process tanks at Davyhulme and Eccles wastewater treatment works, Manchester, England. Organic matter was removed from samples by centrifugation at 3,000*g* for 30 min. 10-ml aliquots of supernatant were filtered (0.22 μm pore size), before being combined with 10 ml of double-strength TSB and 100 μl of exponentially growing bacterial cultures, followed by incubation at 37°C, in an orbital incubator at 150 rpm for 24 h. Bacterial debris were removed by centrifugation (3,000*g*, 30 min), and supernatants were filtered (0.22 μm) and stored at 4°C. This supernatant was used to check the presence of lytic phages using the double-agar overlay method (Kropinski et al., 2009). Isolated single plaques were picked into SM buffer (50 mM Tris-HCl, 8 mM MgSO_4_, 100 mM NaCl, and 0.01% gelatin, pH 7.5) in sterile distilled water, and successive rounds of single plaque purification were carried out until purified plaques were obtained. Purified phage suspensions were maintained at 4°C. *S. carnosus* strain TM300H was used for phage propagation whenever possible; however, for some phages, the methicillin-sensitive *S. aureus* isolate D329 was used (**Table 1**).

Phage host range was determined by spot test, 100 μl of log phase bacterial culture was mixed with 10 ml soft agar, the mixture poured onto 10 ml TSA plates and 10 μl phage lysate (~106 pfu/ml) spotted onto the plate prior to overnight incubation at 37°C. Bacterial strains were classed as wholly sensitive to a particular phage if spot test resulted in a clear plaque, intermediately sensitive if plaques showed evidence of clearing but were hazy or turbid and resistant if no clearing was present. All host range assays were performed in triplicate.

A collection of 32 uncharacterized *S. aureus* phages collected in previous studies was also included in this study.

### *In Vitro* Growth Experiments

#### Growth Kinetics in Planktonic Culture

The growth rate of each bacterial isolate in liquid culture was studied in 96-well flat-bottomed microtiter plates by measuring absorbance of each well using the method of Alves et al. (2014). Briefly, bacterial growth was measured by absorbance (600 nm) over 19 h at 37°C, with shaking, using a microplate reader (FLUOstar Omega, BMG LABTECH). The plate reader provided absorbance data points every 180 s following a 10-s agitation at 200 rpm. Data points after every 30 min were used for analysis.

#### Time-Kill Assays

Time-kill assays were performed to determine the sensitivity of planktonic bacterial cells to phage infection and to investigate the frequency of phage-resistant bacterial mutants using the method described in Alves et al. (2014). Briefly, 200 µl of 1:100 dilutions of overnight bacterial cultures were added to wells of 96-well microtiter plates. Dilutions were made using TSB. After 2 h of incubation at 37°C, phage lysate, at an MOI of 0.1, was added, and the microplates were incubated for a further 17 h. Experiments were performed in triplicate.

#### Formation and Treatment of *S. aureus* Biofilms

Biofilm assays followed a standard 96-well plate method as described previously (Alves et al., 2014). Briefly, 200 µl of 1:100 dilutions of overnight bacterial culture, made using TSB supplemented with 1% D-(+)-glucose (TSBg), were added to microtiter plates. Microtiter plates with lids were sealed with Parafilm were wrapped in moistened paper towel, then placed in a sealed plastic box to maintain humidity. Plates were incubated at 37°C for 48 h without agitation to allow biofilm formation. After 24 h, 50 μl of spent medium was withdrawn and replaced with 50 μl of fresh TSBg. Plates were then incubated for a further 24 h at 37°C. Biofilms were washed three times with PBS before air drying and staining with 0.1% (w/v) crystal violet (CV). Stained biofilms were rinsed with PBS, air dried then solubilized in 200 μl of 30% (v/v) glacial acetic acid. Biofilm mass was measured spectrophotometrically using a FLUOstar plate reader at absorbance of 590 nm. Enumeration of *S. aureus* cells recovered from 48-h biofilms was performed by washing with PBS to remove non-adherent bacteria and residual media. Biofilms were then resuspended in 200-µl PBS and serially diluted, with 100 µl spread on TSA plates to determine the CFU for each isolate.

48-h biofilms were treated with 200 μl of diluted phage lysate at two different MOIs, of 1.0 and 0.1. Quantification of biofilm biomass and viable cell counts following exposure to phage for 6 and 24 h was performed as described above.

### Phage Genome Sequencing

#### Isolation of Phage Genomic DNA

Phage genomic DNA was extracted by a phenol/chloroform/isoamyl alcohol (25:24:1 [v/v]) method using 1.5 ml of lysate (107 to 109 pfu/ml). Lysates were centrifuged at 10,000*g* for 10 min at 4°C, and 1-ml supernatant was transferred into a fresh microfuge tube and treated with DNase I (10 μl of 1 mg/ml DNase I) and RNase A (4 μl of 12.5 mg/ml RNase A). 1 ml of phenol (pH 10) was added to each tube, before vortexing for 30 s, and centrifugation at 10,000*g* for 10 min at 4°C. The aqueous layer was removed to a fresh tube and 1 ml phenol/chloroform/isoamylalcohol (25:24:1) was added, vortexed for 30 s, and centrifuged at 10,000*g* for a further 10 min at 4°C. The phenol/chloroform/isoamylalcohol step was then repeated before DNA was precipitated with two volumes of ice-cold absolute ethanol and 1/10 volume of 7.5 M ammonium acetate, and stored at −20°C overnight. Samples were centrifuged at 10,000*g* for 20 min at 4°C, and DNA pellets were washed twice with 1 ml 70% ethanol (v/v), then resuspended in 100 μl nuclease-free water.

### Whole-Genome Sequencing

Libraries of the selected phage DNA samples (input DNA 0.2 ng/μl) were prepared using the Illumina NexteraXT DNA Sample Preparation Kit following manufacturer’s instructions. Sequencing of phage DNA (paired-end 2 × 150 high output) was carried out using the Illumina NextSeq500 platform at Manchester Metropolitan University, UK.

#### Genome Assembly, Annotation, and Comparison

Sequence reads were assembled using SPAdes v3.11 (Bankevich et al., 2012). All phage assemblies resulted in a single large contig plus a number of small repeats. The largest contig and their coverage were assessed and visualized using Bandage (Wick et al., 2015), individual genome assemblies were analyzed using Artemis (Rutherford et al., 2000), and the largest scaffolds were compared to the similarity of previously sequenced genomes using BLASTN. Based on the similarity between our query sequences and the top hits (closely related genomes) identified using BLASTn, genome assemblies of all related phage infecting *S. aureus* were retrieved from GenBank (https://www.ncbi.nlm.nih.gov/nuccore) and the European Nucleotide Archive (ENA) databases in April 2021 to achieve a final collection of 122 phage genomes.

Genomes were annotated with PROKKA v1.14.6 (Seemann, 2014) using a custom *Caudovirales* gene database (Michniewski et al., 2019). Neighbor-joining trees were constructed using min-hash distances implemented in Mashtree (Katz et al., 2019). Genome comparisons were made using min-hash implemented in MASH (Ondov et al., 2016), and the pan-genome analysis tool Roary v3.13.0 (Page et al., 2015) was used to assess the number of genes shared by each genome (Page et al., 2015). Phylogenetic trees were constructed using Archaeopteryx v0.9929 (https://sites.google.com/site/cmzmasek/home/software/archaeopteryx).

### Statistical Analysis

Planktonic and biofilm experiments were performed with a minimum of three replicates, and these values were used to plot mean ± standard deviation. Statistical analysis was performed using GraphPad Prism Version 7.0 software package, data were analyzed as an ordinary one-way analysis of variance (ANOVA) and Sidak’s multiple comparison test to determine significance of results. Results were taken as significantly different by a *p* value of < 0.05 unless otherwise stated.

## Results

### Phage Isolation

The modified *S. carnosus* strain TM300H and *S. aureus* strain D329 were used to isolate and propagate 46 phages from 150 filtered sewage samples over a period of several months. Plaques were all small in size with most being <1 mm in diameter (*n*=39) and the largest being 2 mm. 39 of 46 phages were propagated on TM300H; however, the remainder (EW20, EW29, EW30, EW41, and EW44-46) could not reliably infect this strain and were propagated on *S. aureus* D239 instead. Phages were named in accordance with recent guidance on nomenclature with the designations vB_SauM_EW1 to vB_SauM_EW46 (Adriaenssens and Brister, 2017) and are henceforth referred to as EW1, EW2 … EW46. The 32 phages isolated previously were propagated on modified TM300H and were named EW47 to EW78.

### Host Range

**Figure 1:**
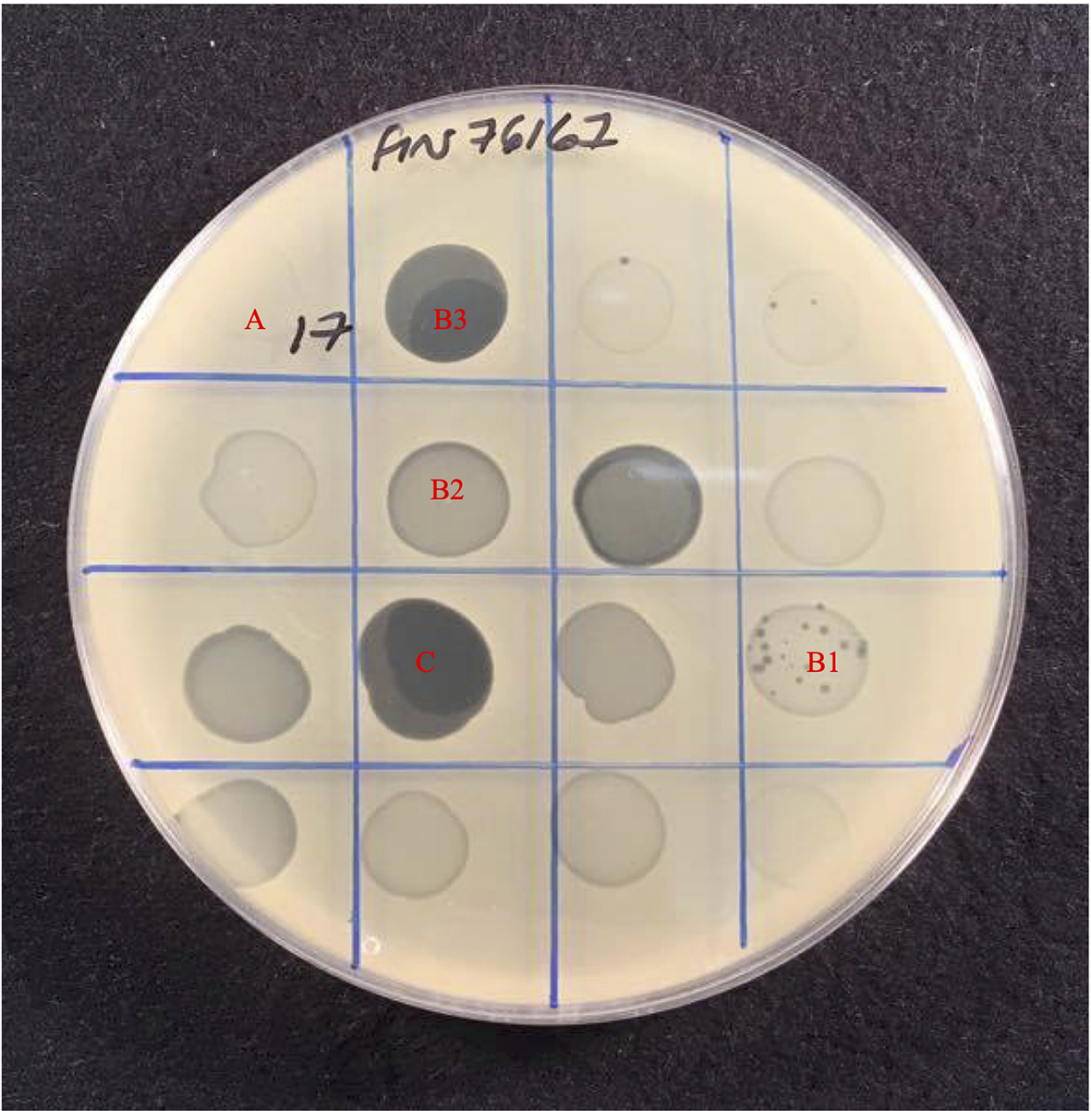
Host range assay showing effect of 16 phages against *S. aureus strain* Fin76167. Plaque formation was scored based on the level of clearing, **(A)** Resistant with no disturbance to lawn, **(B)** Intermediate-sensitivity varied from, B1 Few plaques with slight disturbance to lawn, B2 Substantial turbidity throughout clear zone, B3 High degree of clearing of with numerous mutant colonies present, to **(C)** Sensitive with complete clearing of bacterial lawn.

The host range of the 78 phages was determined by spot test of lysates against 185 *S. aureus* isolates (**Table 2**) in agar overlays. Bacterial strains were classified as sensitive, intermediately sensitive, or resistant, depending on plaque morphology — examples of these are shown in **Figure 1**. The majority exhibited a broad host range phenotype with 40 of our 78 phage capable of infecting over 90% of isolates as determined by their sensitivity or intermediate-sensitivity to phages in this assay (**Table 2**). Fifteen of these were capable of disrupting the growth of over 95% (178/185) of isolates. Phages EW70 and EW71 had the broadest host range and were capable of infecting 184 of the 185 isolates. Of the 184 isolates, they could infect 96 (53%), and 101 (55%) were fully susceptible, respectively, that is, they produced distinct clear plaques. The only phage they could not infect was a mutant of isolate MRSA252 generated in a previous study during growth of the isolate in liquid culture with phage K (Alves et al., 2014).

**Table 2:** Percentage coverage of EW phage against 185 *S. aureus* isolates.

Phage	Isolates Resistant	Isolates Intermediate	Isolates Sensitive	Coverage	Phage	Isolates Resistant	Isolates Intermediate	Isolates Sensitive	Coverage
EW1	80	104	1	56.76%	EW40	17	158	10	90.81%
EW2	80	104	1	56.76%	EW41	6	124	54	96.76%
EW3	27	149	9	85.41%	EW42	6	163	16	96.76%
EW4	59	124	2	68.11%	EW43	11	159	15	94.05%
EW5	26	153	6	85.95%	EW44	44	112	29	76.22%
EW6	42	137	6	77.30%	EW45	36	147	2	80.54%
EW7	48	117	20	74.05%	EW46	65	116	4	64.86%
EW8	163	21	1	11.89%	EW47	123	62	0	33.51%
EW9	70	110	5	62.16%	EW48	60	122	3	67.57%
EW10	59	117	9	68.11%	EW49	14	147	24	92.43%
EW11	97	83	5	47.57%	EW50	139	41	5	24.86%
EW12	71	112	2	61.62%	EW51	15	151	19	91.89%
EW13	82	100	3	55.68%	EW52	6	152	27	96.76%
EW14	80	101	4	56.76%	EW53	27	149	9	85.41%
EW15	7	119	59	96.22%	EW54	16	94	75	91.35%
EW16	69	57	59	62.70%	EW55	76	96	13	58.92%
EW17	76	52	57	58.92%	EW56	19	119	47	89.73%
EW18	3	98	84	98.38%	EW57	16	116	53	91.35%
EW19	70	59	56	62.16%	EW58	14	107	64	92.43%
EW20	71	60	54	61.62%	EW59	13	101	71	92.97%
EW21	18	109	58	90.27%	EW60	12	84	89	93.51%
EW22	26	102	57	85.95%	EW61	13	99	73	92.97%
EW23	37	119	29	80.00%	EW62	12	94	79	93.51%
EW24	30	139	16	83.78%	EW63	12	99	74	93.51%
EW25	46	130	9	75.14%	EW64	11	97	77	94.05%
EW26	7	116	62	96.22%	EW65	13	107	65	92.97%
EW27	5	126	54	97.30%	EW66	12	96	77	93.51%
EW28	19	146	20	89.73%	EW67	11	94	80	94.05%
EW29	6	142	37	96.76%	EW68	11	97	77	94.05%
EW30	16	158	11	91.35%	EW69	10	103	72	94.59%
EW31	19	154	12	89.73%	EW70	1	88	96	99.46%
EW32	54	122	9	70.81%	EW71	1	83	101	99.46%
EW33	19	160	6	89.73%	EW72	5	91	89	97.30%
EW34	36	138	11	80.54%	EW73	13	95	77	92.97%
EW35	4	169	12	97.84%	EW74	2	76	107	98.92%
EW36	7	164	14	96.22%	EW75	8	81	96	95.68%
EW37	14	167	4	92.43%	EW76	10	83	92	94.59%
EW38	36	148	1	80.54%	EW77	13	89	83	92.97%
EW39	142	41	2	23.24%	EW78	10	85	90	94.59%

The phage host range is scored as resistant, intermediate, and susceptible based on the level of clearing on host overlays. Percentage coverage is the cumulative number of isolates that displayed intermediate and sensitive susceptibility to phage.

Coloured shading has been used to indicate level of resistance (red), intermediate-sensitivity (orange), and sensitivity of the isolate collection to phage infection.

### Time Kill Assays in Planktonic Culture

To investigate the dynamics of phages and their hosts in liquid culture, we quantified the ability of phages to reduce bacterial numbers in broth cultures and observed any phage-resistant mutant emergence. We selected ten of the fourteen phages that were able to infect > 96% of isolates tested, for time kill experiments (from **Table 2**). These were phages EW15, EW18, EW27, EW29, EW36, EW41, EW52, EW71, EW72, and EW74. Suspensions of these ten phages were tested against TM300H, D329, MRSA252, and 15981. These isolates were chosen as they include the two propagating bacteria used, MRSA252, a representative of a major MRSA lineage (CC30) and the first genome sequenced MRSA isolate (Holden et al., 2004) and the well-studied abundant biofilm producing isolate 15981. Phages were introduced to growing cultures to achieve a multiplicity of infection (MOI) of 0.1, and incubated for 19 h with OD_600_ readings taken every 3 min.

**Figure 2:**
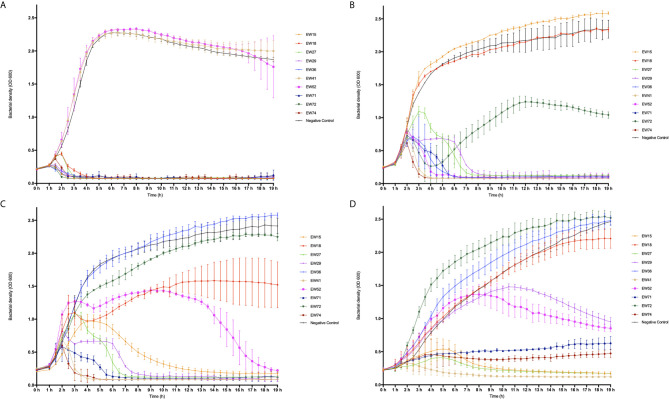
Time-kill curve of four mid-exponential phase planktonic *S. aureus* strains by ten different phages at a multiplicity of infection (MOI) of 0.1. Absorbance readings at 600 nm were taken using a plate reader every 30 min for 19 h while shaking at 37°C, three independent experiments were performed in total. **(A)** Modified *S. carnosus* isolate TM300H; **(B)** *S*. *aureus* isolate D329, **(C)** *S*. *aureus* isolate MRSA252, and **(D)** *S*. *aureus* isolate 15981.

Both EW41 and EW52, propagated on D329 were the only phage ineffective against TM300H in this planktonic culture assay. The remaining eight phages were successful in reducing the growth of TM300H within 4 h following their introduction and they prevented any observable growth of phage-resistant mutants after 19 h (**Figure 2A**). With isolate D329, phages EW27 and EW29 initially took an hour longer than other phage before having any effect on the host as seen in **Figure 2B**. However, both EW27 and EW29 effectively reduced the growth of D329 after 4 and 6 h, respectively, while preventing the emergence of phage-resistant mutants. As for phage EW72, it was unsuccessful at depleting bacterial numbers before phage-resistant mutants emerged after 5 h, although this had an effect on the growth rate of D329 when compared with controls. Interestingly, an increase in bacterial density compared with the control was observed in D329 following addition of EW36. Individual growth phases appear less clearly defined with MRSA252 when challenged with phage (**Figure 2C**), which is also observed with strain 15981 (**Figure 2D**). It is clear that a number of phages could not effectively reduce bacterial numbers before resistant mutants emerge. A reduced rate of killing was observed among phage when challenged against other strains compared with their propagating hosts, decreasing bacterial numbers at a much more gradual rate, with some phage such as EW15 taking several hours. Interestingly, phage EW71 and EW74 appeared to have a bacteriostatic effect on strain 15981 with no change in absorbance observed for c. 14 h before slowly increasing (**Figure 2D**).

As observed in D329, EW36 seemed to have a positive effect on the growth of MRSA252 when compared with the control, as a significant increase in OD_600_ with time was observed. The same trend was also observed with EW72 on the growth of 15981, however, only slightly more than the control. Interestingly, EW52 appeared to have a brief positive effect on the growth rate of MRSA252 for a period of 2 h when initially introduced to the wells, followed by a prolonged infection period in which MRSA252 appeared to increase in concentration momentarily, before eventually decreasing in bacterial density below the initial concentration after 15 h. For all three *S. aureus* isolates used, phages that were able to successfully reduce the optical density and prevent bacterial regrowth were able to achieve it within 8 h relative to controls. Nevertheless, there were a number of phages including EW15, EW27, EW29, and EW52 that exhibited a lower degree of bacteriolytic ability against their hosts, taking up to ~13 h to have any inhibitory and bactericidal effect on the bacteria. Isolate 15981 rapidly evolved resistance to phages EW18 and EW52. However, phage EW52 was successful in reducing bacterial density eventually, whereas phage EW18, moderately reduced growth of 15981 but elicited resistant mutants. Strain 15981 was found to be the most resistant to phage infection killing in this assay. Reductions to growth were at a much lower rate compared to other hosts and the appearance of phage-resistance was observed in each experiment. Phage EW41 was the most effective at inhibiting the growth of all three *S. aureus* isolates, decreasing cell densities rapidly, within 2 h following phage application. However, as EW41 was isolated and propagated on D329 because it could not be propagated on TM300H, it is not surprising that it had little or no effect on TM300H in this assay.

### *S. aureus* Biofilms

The two most common MRSA lineages in the UK have MLST sequence types (ST) 22 and 36 and these currently have global distributions (Holden et al., 2004; Holden et al., 2013). We selected isolates from these lineages to examine their biofilm forming properties and susceptibility to phages as these genotypes are the most common in our collection. We compared the biofilm densities produced by 43 ST22, and 27 ST36 isolates after 48 h by measurement of absorbance at OD_590_ following CV staining. We found considerable variation within isolates of these genotypes (**Supplementary Figure 1**) with an obvious dichotomy between relatively little biofilm produced by most ST22 isolates compared to those of ST36. This can be seen by visual inspection of stained biofilms from the four most proficient biofilm producing isolates from each ST with ST36 isolate biofilms being generally darker stained than those of ST22 (**Supplementary Figure 2**).

We used the same ST22 isolates; ARI10, WW44936, 1018.07, and HO5322054809 and ST36 isolates; 07.1696.F, 06.9570.L, 07.1227.Z, and 07.2880.V (see **Table 1** for details on isolates) to examine the relationship between biofilm biomass and viable cell count. We compared OD_590_ readings of CV-stained 48 h mature biofilms for these eight isolates and compared these to viable cell counts. **Supplementary Figure 3** shows that the variation in biofilm biomass between strongest and weakest biofilm formers does not necessarily correlate to the number of viable cells present. All isolates had approximately similar numbers of cells in their biofilms but there was markedly greater variation within OD_590_ readings for some isolates. ST22 isolates ARI10 and WW936 produced significantly more CV-stained biofilm than 1018.07 and 370.07 and similarly for ST36 isolates 07.1696.F and 06.9570.L produced much more CV-stained biofilm than the other two isolates of this genotype.

ST22 strains displayed a propensity to form moderately adhered biofilms that had significantly lower ODs than the best ST36 biofilm formers, yet they had consistently higher cell counts — similar to those values observed from ST36 isolates. The biofilms produced by *S. aureus* were found at the air-biofilm interface and as large aggregates at the solid-liquid interface at the base of microtiter plate wells.

### Effect of Phages on 48 h Biofilms

We selected four phages (EW27, EW36, EW41, and EW71) for analysis of biofilm reduction on the basis of; i) their broad host range against study isolates (**Table 2**) and ii) rapidly lytic characteristics in planktonic culture and observed lack of resistant-mutant selection (**Figure 2**). Each of these four phages was added to 48 h biofilms at an MOI of 0.1 or 1.0 and viable cell counts and OD_600_ readings of CV-stained biofilms were performed after 6 and 24 h following phage application. Biofilm readings for each phage and corresponding viable cell counts are presented in **Supplementary Figures 4**–**7** and biofilm readings summarized in **Table 3**.

**Table 3:** Summary table showing the relative difference in biofilm reduction of study phage at two multiplicities of infection (MOI) against four ST22 and four ST36 isolates.

	ST22	OD590	MOI 1	EW27MOI 0.1	MOI 1	EW36MOI 0.1	MOI 1	EW41MOI 0.1	MOI 1	EW71MOI 0.1
	**ARI 10**	1.2034	-44%	-61%	-47%	-63%	-80%	-85%	-84%	-87%
6 h	**W449 36**	1.1929	-71%	-78%	-77%	-78%	-22%	-19%	-75%	-68%
	**1018.07**	0.5941	-59%	-74%	-58%	-72%	-59%	-70%	-67%	-73%
	**370.07**	0.4381	-43%	-68%	-52%	-63%	-43%	-69%	-63%	-76%
	**ARI 10**	1.2034	-58%	-55%	-81%	-82%	-82%	-83%	-87%	-86%
24 h	**W449 36**	1.1929	-68%	-77%	-82%	-85%	-85%	-86%	-84%	-84%
	**1018.07**	0.5941	-20%	-49%	-68%	-69%	-69%	-70%	-76%	-74%
	**370.07**	0.4381	-25%	-43%	-59%	-68%	-63%	-66%	-78%	-75%
	ST36	OD590	MOI 1	EW27MOI 0.1	MOI 1	EW36MOI 0.1	MOI 1	EW41MOI 0.1	MOI 1	EW71MOI 0.1
	**07.1696.F**	3.0191	-89%	-89%	-79%	-79%	-90%	-86%	-95%	-95%
6 h	**06.9570.L**	2.7274	-75%	-60%	-81%	-80%	-71%	-74%	-91%	-92%
	**BTN 2172**	1.0997	-74%	-72%	-76%	-73%	-86%	-85%	-83%	-83%
	**07.2496.L**	0.7536	-64%	-69%	-20%	-29%	-70%	-70%	-81%	-79%
	**07.1696.F**	3.0191	-77%	-80%	-88%	-90%	-86%	-89%	-89%	-95%
24 h	**06.9570.L**	2.7274	-25%	-51%	-89%	-90%	-59%	-84%	-90%	-92%
	**BTN 2172**	1.0997	-30%	-57%	-73%	-78%	-83%	-68%	-77%	-83%
	**07.2496.L**	0.7536	-40%	-66%	-56%	-60%	-36%	-66%	-59%	-79%

48 h biofilms were challenged with phage for 6 and 24 h, percentages are based on control values.

Colour used to indicate different treatments.

#### EW27

Viable cell counts recovered from each EW27 phage-treated biofilm for all ST22 and ST36 isolates except for isolate 07.2496.L, were significantly reduced (*p* < 0.001) following a 6-h exposure to EW27 when compared to untreated biofilm controls (**Table 3** and **Supplementary Figure 4**). However, following an initial decrease in CFU/ml after 6 h, an increase in bacterial concentration can be seen across all phage-treated ST22 isolates after 24 h, suggesting that resistance to phage had occurred within that time. There was no significant difference between CFU counts from wells treated with phage for 6 and 24 h (*p* < 0.05). When considering the overall biofilm biomass following phage exposure and CV staining, results revealed phage EW27 was highly effective at reducing the biofilms produced by all ST22 and ST36 strains. For EW27 treated ST36 isolates, biofilm biomass significantly increased in isolates 07.1696.F, 06.9570.L, and BTN 2172 using both MOI 1 and 0.1 (*p* < 0.05), despite a minor reduction in bacterial numbers after 24-h treatments compared to 6 h. Following treatment of EW27 after both timepoints, EW27 at a MOI of 0.1 proved to be the most effective at both reducing bacterial cells and biofilm biomass for almost all ST22 and ST36 strains.

#### EW36

Phage EW36 produced significant reductions in biofilm biomass for all study isolates except for 07.2496.L at both MOIs (*p* < 0.01), with MOI 0.1 proving to be most effective (**Table 3** and **Supplementary Figure 5**). For both ST22 and ST36, no increase to biofilm density was observed from 6 to 24 h, suggesting that phage EW36 successfully disrupted biofilms preventing regrowth. This is further supported by the greater reduction in viable cell counts when biofilms were treated for 24 h. Interestingly, the populations of viable bacteria recovered from each biofilm produced by the four ST22 and ST36 isolates were found to be higher in wells treated by phage EW36 at MOI 0.1, despite producing lower absorbance readings than biofilms treated with a higher titer of phage at a MOI 1. EW36 was able to reduce viable cell numbers for both ST22 and ST36 isolates by at least one-log after 6 h and two-logs after 24 h at an MOI 0.1. With biofilms treated at an MOI 1, two-log reductions were observed after 6 h and three-log reductions after 24 h.

#### EW41

Significant reductions (*p* < 0.01) in biofilm biomass were observed for all ST22 and ST36 isolates tested with phage EW41 except for isolate 370.07 where two-log reductions in cells recovered and 60% to 93% reductions in biofilm biomass were observed after 6 h treatment (**Table 3** and **Supplementary Figure 6**). Interestingly, phage EW41 had the least effect in reducing biofilm biomass of isolate W449 36 after 6 h — reducing it by roughly 22% at an MOI 1 and 19% at an MOI 0.1; however, viable cell counts were relative to all other isolates and two-log reductions were observed across both time points. Furthermore, biofilm biomass and viable cell counts recovered from the biofilms challenged with EW41 after 24 h produced levels similar to 6 h exposure. Phage EW41 was able to further reduce biofilm levels of W449 36 by ~85% when exposed for 24 h. ST36 biofilms challenged with phage EW41 for 24 h produced higher levels of biofilm biomass and increase in cells recovered by up to one-log when compared to 6 h exposure, suggesting regrowth had occurred within that time. Across all ST22 and ST36 isolates, both biofilm biomass and viable cells recovered were consistently lower in wells challenged with EW41 at an MOI 0.1 when compared with MOI 1, although this was not significant.

#### EW71

Phage EW71 was the most effective of the four in reducing biofilm density and viable cell numbers (**Table 3** and **Supplementary Figure 7**). Phage EW71 was effective at reducing (*p* < 0.01) biofilm biomass after 6 h treatment while greatly limiting the amount of regrowth after 24 h. Furthermore, phage EW71 was successful in reducing the number of viable cells by up to three-logs after 6 h, and continued to reduce after 24 h treatment by up to four logs versus controls. Biofilm densities of ST22 following treatment of EW71 after 6 h ranged from 63% to 87% while consequently preventing the regrowth of all four ST22 hosts after 24 h, further reducing biofilm densities. Greater reductions in biofilm densities were also observed when ST36 isolates were challenged with phage EW71 with OD_590_ values reduced by 59 95% after 6 h. Interestingly, a marginal increase in absorbance was observed in across all four ST36 biofilms when exposed to phage for 24 h. However, increases to viable cell counts were only observed for 07.1696.F and 07.2496.L suggesting phage resistance and regrowth had occurred within the two sampling periods. Phage applied to biofilms at an MOI 1 were found to be the most effective at reducing viable cell counts within the biofilm after 6 and 24 h exposures; however, biofilm densities were somewhat higher with this MOI. Even so, biofilm biomasses were approximately similar across the majority of hosts for both 6- and 24-h treatments, except for isolate W449 36; however, this difference was not significant.

### Evaluation of Phage Biofilm Assays

**Table 3** and **Supplementary Figures 4**–**7** show the percentage reduction of *S. aureus* ST22 and ST36 isolate biofilms when challenged by EW27, EW36, EW41, and EW71 at MOIs of 1 and 0.1 after 6 and 24 h. For all ST22 isolates the median reduction in biofilm biomass for MOI 1 and MOI 0.1 after 6 h was 59% and 71% respectively, whereas the median reduction for MOI 1 and MOI 0.1 after 24 h exposure was 72% and 75%, respectively. Whereas, for ST36 isolates, the median biofilm biomass reduction for MOI 1 and MOI 0.1 after 6 h was 80% and 79%, respectively. After 24 h treatment, the median reduction for MOI 1 and MOI 0.1 for ST36 isolates was 75% and 80%, respectively.

Overall, the highest biofilm biomass reductions after 6 and 24 h phage treatments was observed with phages EW41 and EW71 respectively, with an MOI of 0.1. Although significant reductions in biofilm biomass was observed when treated with both MOIs of phage, the greatest reductions across all ST22 and ST36 isolates after 6 and 24 h exposure was achieved when biofilms were treated at an MOI 0.1. Although each phage was able to disperse the biofilms of all study isolates, complete elimination of cells was not observed across any of the hosts at either MOI as cells were recoverable when treated with phages for 6 h and 24 h.

### Phage-Resistant Mutants

**Figure 3:**
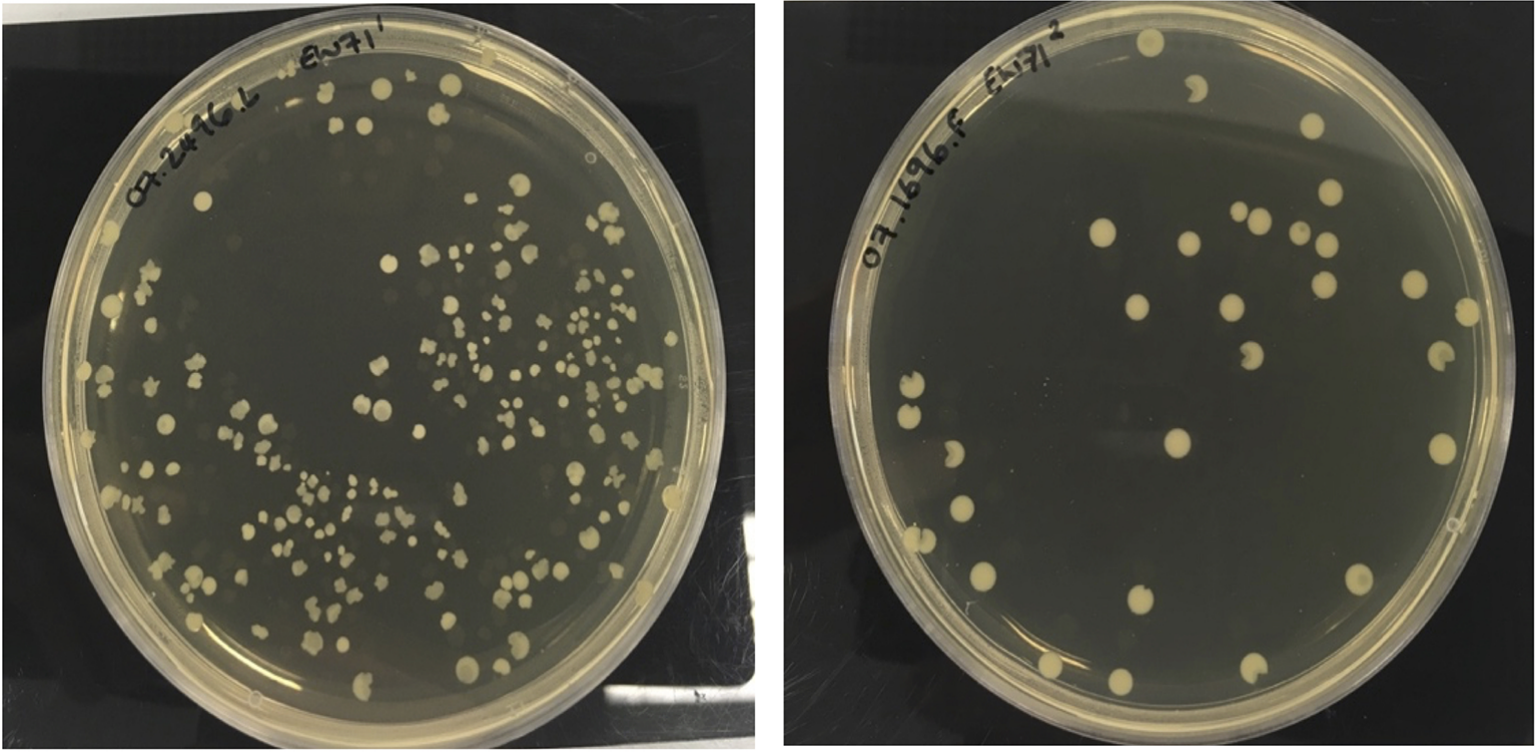
Heterogeneous colony phenotypes produced by *S. aureus* phage-resistant mutants of 07.2496.L and 07.1696.F following exposure to EW71.

The morphology of colonies recovered from phage-treated biofilms were heterogeneous and this was most marked for isolates treated with phage EW71 as shown in **Figure 3**. Phage-resistant mutant isolates recovered from each phage experiment were found to be resistant to all phage upon spot testing on agar overlays.

### Genomics

**Figure 4:**
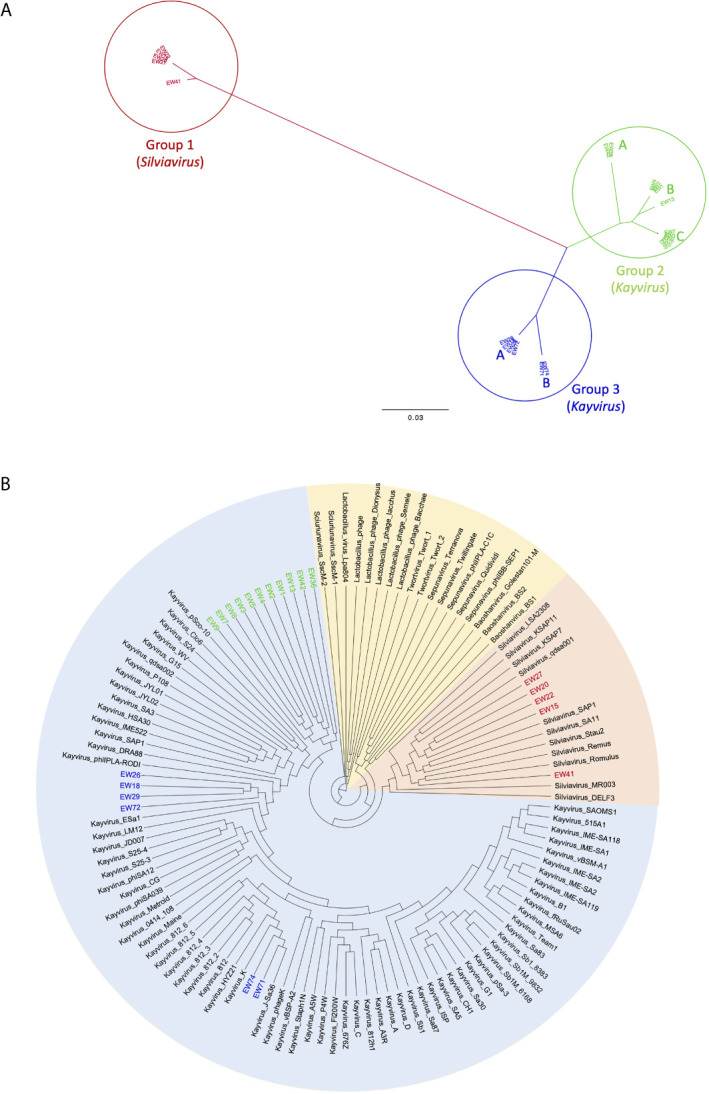
Neighbor-joining dendrograms based upon min-hash (MASH) distances. **(A)** unrooted dendrogram of phages EW1-22 showing three major groupings and subgroupings. **(B)** Circular dendrogram showing the relatedness of the same phages in relation to 100 *Twortvirinae* genome assemblies and the phage genera they represent. Dendrogram is rooted at the midpoint of the longest branch.

We sequenced the genomes of 22 phages with broad host range based upon spot testing results (**Table 2**). All are *Myoviruses* and members of the *Twortvirinae* sub-family of the family *Herelleviridae* based on BLASTN similarity. To further investigate the relatedness and taxonomy of our phages we compared their genomes to the 100 publicly available *Twortvirinae* genomes in Genbank (as of April 2021) using the min-hash algorithm implemented in MASH (Ondov et al., 2016) to generate distance matrices that were used to construct the neighbor-joining dendrograms shown in **Figure 4**. The 22-phage genomes segregated into three main groups, designated 1 3.

#### Group 1

This group contains five phages EW15, EW20, EW22, EW27, and EW41 (shown in red in **Figure 4**). They share very high similarity (MASH distances < 1%) with each other, except for phage EW41 that differs by 1.6% (**Supplementary Table 1**). It also has a slightly smaller genome of 132,999 bp compared to the others which are c.135,800 bp in size. These phages are very similar to, and cluster with 11 staphylococcal phages of the genus *Silviavirus* (**Figure 4B**). They include the broad host-range phages Romulus and Remus, proposed as an ideal candidate phages for human therapy due to their broad host-range and virulence (Vandersteegen et al., 2013).

#### Group 2

The largest group in this study contains 11 phages of the genus *Kayvirus* based on genomic sequence similarity, with genomes that share >95% similarity (*i.e.* < 5% MASH distance) with each other (**Figure 4** and **Supplementary Table 1**). It contains three subgroups A, B, and C, and these are colored green in **Figure 4**. Group 2A contains phages EW36 and EW42 that are 139,881 and 139,874 bp in length, respectively. Group 2B is made up of phages EW1, EW2, EW4 that are very similar (>99%) to each other and EW13 that differs from these by about 1.8% of bases The first three phages have genomes that are 140,906 bp long, about 4 kb shorter than that of EW13 at 145,736 bp. Group 2C comprises phages EW3, EW5, EW6, EW7, and EW9 with genomes of between 141,953 and 143,288 bp. Their genomes are >99.5% similarity to each other. Group 2 phages form a distinct clade on their own in the dendrogram in **Figure 4B** and are most closely related to genomes belonging to phages of the genus *Kayvirus* that include the species “Staphylococcus phage MCE-2014” (Alves et al., 2014) and several unclassified Kayviruses, sharing c.90% sequence similarity. Phage MCE-2014 (also known as DRA88) was first reported in a study where it was used in combination with phage K to treat experimental *S. aureus* biofilms. The phage K mutant of strain MRSA252 used here was derived from this study (**Table 1**).

#### Group 3

This group contains two closely related subgroups pf phages whose genomes differ by c.3%. The four group 3A phages in this group are EW18, EW26, EW29, and EW72 with genomes of 143, 240 to 143,287 bp that are > 99.5% similar to each other. These cluster most closely with database isolates of the genus *Kayvirus* that include the species phiPLA-Rodi whose reference phage, phiIPLA-RODI, has been used in several studies, including those involving *S. aureus* biofilms (Gutierrez et al., 2015; Gonzalez et al., 2017). The genomes of group 3B phages EW71 and EW74 are 139,939 and 139,896 bp in length respectively, and they share >99% sequence similarity with each other and with the genomes of three phages in Genbank from the genus *Kayvirus*. These include phage K (Gill, 2014), the most well known of the staphylococcal phages (**Figure 4B**) that is commonly included in staphylococcal phage preparations (O’Flaherty et al., 2005b).

Overall, the genomes of *Silviavirus* phages of group 1 share about 25% to 26% DNA sequence similarity to those of group 2 and group 3 *Kayvirus* phages. Groups 2 and 3 are more similar with approximately 90% DNA similarity using min-hash distances (**Supplementary Table 1**). **Supplementary Table 2** lists all annotated genes in the 122 *Twortvirinae* studied and shows their presence absence (BLASTP identity > 95%). Group 1 phages had no genes in common with those of groups 2 and 3 using Roary with default parameters. Groups 2 and 3 shared 38 genes common at BLASTP identity > 95% (18% of the 202 genes in the EW1 genome). No known function could be assigned to 33 but the core genes comprised a terminase gene, an intron-encoded endonuclease, a LysM domain-containing protein, a putative DNA repair protein, and a virion component protein.

The host range of group 1 phages as measured by percentage of strains able to be infected (coverage) varied from 61.62% to 97.3%. In group 2 this coverage varied from 56.76% to 96.76% and in group 3 from 96.22% to 99.46% (**Table 2**). All three groups therefore had phages that could infect the great majority, if not all 184 clinical isolates, but not the phage K mutant MRSA252 strain.

## Discussion

Phage therapy has potential for the treatment of many bacterial diseases, but for most bacterial pathogens, the limited host range of lytic phages means that empiric use requires the use of cocktails of different phage strains with varying host ranges and virulence characteristics. Broad host range, highly virulent *S. aureus* phages are relatively easy to isolate, and their use in phage therapy, especially in Georgia and Poland, has been associated with a high degree of success. This study confirms reports of the extremely broad host range of some Myoviridae, especially some of the Kayviruses and Silviaviruses characterized here. Two of these Kayviruses, EW70 and EW71, were able to infect the complete panel of our genetically diverse 185 *S. aureus* isolates in agar overlays that included a very wide range of MRSA and MSSA genotypes. Nine other phages could infect >96% of isolates, and these comprised three Silviaviruses, two Kayviruses of a different clade from EW70 and EW71, as well as four others that are closely related to EW70 and EW71. The broad host range of *S. aureus* Myoviruses is in part explained by their sharing a common receptor that has been found to be the backbone of cell wall teichoic acids (Xia et al., 2011; Winstel et al., 2013).

In our planktonic assays, all 10 phages were able to infect at least one of the four *S. aureus* isolates tested, and five were able to infect and significantly reduce the growth of two. EW41 was found to be the most effective under planktonic conditions, as it immediately reduced bacterial cell numbers preventing the regrowth of all three *S. aureus* isolates, yet it had no effect on the modified *S. carnosus* isolate. As it was propagated on *S. aureus* strain D239 host, its specificity may be more limited compared to other phages in infecting coagulase-negative staphylococci. Four phages had no effect on the growth of at least one of the three *S. aureus* hosts in liquid culture. However, phages that were effective were able to prevent the appearance of resistant mutants throughout the duration of the experiment. When resistance mutants were observed, the growth rate and presumably, fitness of these phage-resistant cells was clearly affected and did not recover to the levels achieved by uninfected controls. This suggests that resistance to phage infection was at the expense of growth capacity (Middelboe, 2000; Avrani and Lindell, 2015). Emergence of spontaneous phage resistance can involve selection of sub-populations with altered receptor structures that in *S. aureus* includes wall techoic acid (Hall et al., 2011; Xia et al., 2011; Avrani and Lindell, 2015). Fitness costs associated when acquiring phage-resistance, can cause a variety of structural and morphological changes (Inal, 2003; Ormala and Jalasvuori, 2013). One approach is concealing surface receptors that phages used as docking sites to adsorb to their hosts; however, these sites are often used for the uptake of nutrients (Mizoguchi et al., 2003), thus possibly limiting their growth and virulence which may be why cell numbers were able to recover after 24 h treatment compared with 6 h, yet biofilm densities remained considerably low.

When applied at low concentrations (MOI 0.1), phage must infect and replicate enough to increase their number to surpass the rate of replication for the bacterial host. This would explain why most host isolates continued to grow for at least 1 h following introduction. In similar studies comparing phage infection at various MOIs (Abedon, 1992; Beeton et al., 2015; Cui et al., 2017a), the greater the MOI the more effective the phage was in the study, which presumably is largely because of the increased rate of phage collisions and infections, thus leading to higher densities in viral progeny in a shorter time frame.

The four *S. aureus* phages used in biofilm studies were selected based on their lytic potential in spot plate assays and in liquid culture. All four exhibited generally high efficacy in effectively reducing biofilm biomass and cell numbers of each *S. aureus* isolate after 6 and 24 h. Cell regrowth was detected following 24 h infection with phage compared to 6 h by at least one of the hosts suggesting growth of phage-resistant mutants had occurred, however this was at the expense of biofilm regrowth. These observations were similar to those reported in a previous study of *S. aureus* biofilm (Melo et al., 2018). The observation of biofilm regrowth and phage resistance still remains a major issue and is something regularly observed in biofilms when challenged by single lytic phage that promotes mutant selection (Drilling et al., 2014). This necessitates a phage combination approach using phage cocktails or co-administration with antibiotics to prevent the emergence of phage-resistant mutant bacteria. Previous studies have made use of the disruptive ability of phage to reduce biofilm structures produced by *S. aureus* and reduce bacterial populations enough to facilitate the penetration of antibiotics and eradicate infection (Tkhilaishvili et al., 2018; Dickey and Perrot, 2019). Previous evidence suggests that the phage resistance phenotype increases sensitivity to antibiotics and also results in a loss of fitness (Leon and Bastias, 2015). Additionally, the application of phage cocktails consisting of multiple polyvalent phage that target different receptor proteins to prevent multi-resistance, but also increase the rate of killing, thus greatly reducing the probability of hosts acquiring resistance to phage (Gu et al., 2012). The anti-biofilm capabilities and broad host range demonstrated by the four study phage make them promising candidates for possible future combination studies.

Previous phage/host studies have demonstrated that by increasing the concentration of phage-to-bacteria (MOI), which, essentially increases the rate of collisions between phage and biofilm cells leads to increased rate of bacterial killing (Gupta and Prasad, 2011; Lopes et al., 2018). Additionally, greater reductions could have been facilitated by direct bacterial lysis the lysis from without effect. However there was no significant difference between MOI values, suggesting that an increased phage-to-bacteria ratio offered no advantages in reducing biofilm biomass, as described previously (Lopes et al., 2018). Overall, reductions in biofilm biomass (OD_590_) were generally higher in biofilms treated at an MOI 0.1 (compared to 1) using any of the four phage after both 6 and 24 h treatments. The effectiveness of the low MOI demonstrates the self-perpetuating nature of lytic phage to proliferate in number, therefore only requiring small initial dosing.

Compared to the characteristic smooth, round colonies phenotypes typically produced by *S. aureus*, the recovery of heterogenous morphotypes produced by phage-resistant derivatives following phage exposure were regularly detected during this study, although most commonly observed in biofilms treated with EW71. Similar irregular-shaped colonies have been reported in previous studies and are thought to be caused by a subpopulation within a colony that has reverted back to a phage-sensitive phenotype, subsequently leading to cell death as the colony forms (Mizoguchi et al., 2003; O’Flynn et al., 2007; Kocharunchitt et al., 2009). However, the observation of “pacman”-like colonies, as seen here, has not been as well documented and warrants further investigation.

A major consideration in producing phages for human therapy is the possible presence of induced prophage from propagating host bacteria. *S. aureus* isolates typically harbor several prophages in their genomes and these mediate horizontal gene transfer and contain virulence genes such as toxins (Xia and Wolz, 2014). In this study, we found that most phages could be propagated on an avirulent *S. carnosus* strain that could be used to increase the safety of staphylococcal phage therapeutics in future GMP manufacturing if used in place of potentially virulent *S. aureus* hosts.

## Data Availability Statement

The datasets presented in this study can be found in online repositories. The names of the repository/repositories and accession number(s) can be found below: https://www.ncbi.nlm.nih.gov/genbank/, GCA_011207355, GCA_011207615, GCA_011207935, GCA_011208035, GCA_011208175, GCA_011208205, GCA_011208235, GCA_011208435, GCA_011207435, GCA_011207495, GCA_011207545, GCA_011207665, GCA_011207715, GCA_011207785, GCA_011207815, GCA_011207845, GCA_011207985, MT080595, GCA_011208105, GCA_011208285, GCA_011208355, and GCA_01120837.

## Author Contributions

ME conceived this study. ME, EW, JR, GX, AM, RR, and SM designed experimental procedures. EW, ME, JR, SM, and RR performed the experiments, analyzed and curated the data. EW and ME assembled the phage collection. ME, EW, JR, AM, and GX wrote the manuscript. All authors contributed to the article and approved the submitted version.

## Funding

EW was funded by a PhD studentship from Faculty of Science and Engineering, Manchester Metropolitan University.

## Conflict of Interest

The authors declare that the research was conducted in the absence of any commercial or financial relationships that could be construed as a potential conflict of interest.

## Supplementary Material

Supplementary Figure 1 Biofilm formation of **(A)** 43 *S. aureus* ST22; and **(B)** 27 ST36 isolates grown in tissue culture microtiter plates over 48 h at 37°C. Biofilms densities produced were assessed following crystal violet staining, dissolved in 30% acetic acid, and measured at an OD590. Each assay was performed in triplicate, data presented as mean values (± standard deviation).

Supplementary Figure 2 Variation in biofilm production by ST22 **(A)** and ST36 **(B)** isolates after 48 h incubation in TSB supplemented with 1% D-(+)-glucose. Biofilms were visualized following staining with 0.1% crystal violet.

Supplementary Figure 3 Comparison of biofilm densities of eight *S. aureus* isolates assessed by CV staining **(A)** and corresponding viable cell counts **(B)**.

Supplementary Figure 4 Effect of phage EW27 on mature biofilms of S. aureus ST22 **(A)** and ST36 isolates **(B)**. Static biofilms were initially grown in tissue-culture microtiter plates for 48 hand challenged with EW36 at a multiplicity of infection of 1 and 0.1 for a period of 6 and 24 h. **(A)** Biofilms initially stained with crystal violet and optical density was measured at an absorbance of 590 nm. **(B)** Viable cells were recovered from phage treated wells by scratching and dislodging the biofilms from the surface plate wells and plated out in triplicate. Each assay was performed in triplicate, data presented as mean values (± standard deviation).

Supplementary Figure 5 Effect of phage EW36 on mature biofilms of *S. aureus* ST22 **(A)** and ST36 isolates **(B).** Static biofilms were initially grown in tissue-culture microtiter plates for 48 h and challenged with EW36 at a multiplicity of infection of 1 and 0.1 for a period of 6 and 24 h. Top Biofilms initially stained with crystal violet and optical density was measured at an absorbance of 590 nm. Bottom Viable cells were recovered from phage treated wells by scratching and dislodging the biofilms from the surface plate wells and plated out in triplicate. Each assay was performed in triplicate, data presented as mean values (± standard deviation).

Supplementary Figure 6 Effect of phage EW41 on mature biofilms of *S. aureus* ST22 **(A)** and ST36 isolates **(B)**. Static biofilms were initially grown in tissue-culture microtiter plates for 48 h and challenged with EW36 at a multiplicity of infection of 1 and 0.1 for a period of 6 and 24 h. Top Biofilms initially stained with crystal violet and optical density was measured at an absorbance of 590 nm. Bottom Viable cells were recovered from phage treated wells by scratching and dislodging the biofilms from the surface plate wells and plated out in triplicate. Each assay was performed in triplicate, data presented as mean values (± standard deviation).

Supplementary Figure 7 Effect of phage EW71 on mature biofilms of *S. aureus* ST22 **(A)** and ST36 isolates **(B).** Static biofilms were initially grown in tissue-culture microtiter plates for 48 h and challenged with EW36 at a multiplicity of infection of 1 and 0.1 for a period of 6 and 24 h. Top Biofilms initially stained with crystal violet and optical density was measured at an absorbance of 590 nm. Bottom Viable cells were recovered from phage treated wells by scratching and dislodging the biofilms from the surface plate wells and plated out in triplicate. Each assay was performed in triplicate, data presented as mean values (± standard deviation).

Supplementary Table 1 Min-hash distances of study phage genomes expressed as percent.

Supplementary Table 2 Gene presence or absence in 122 study phage genomes (BLASTP>95%).4L2_Filename: fcimb.2021.698909_styled.docx
