# Supplementary figures and images for: Phenotypic and Genotypic Characterization of Novel Polyvalent Bacteriophages With Potent In Vitro Activity Against an International Collection of Genetically Diverse Staphylococcus aureus
Source: Front Cell Infect Microbiol. 2021 Jul 6;11:698909. doi: 10.3389/fcimb.2021.698909 (PMC8290860; doi:10.3389/fcimb.2021.698909)

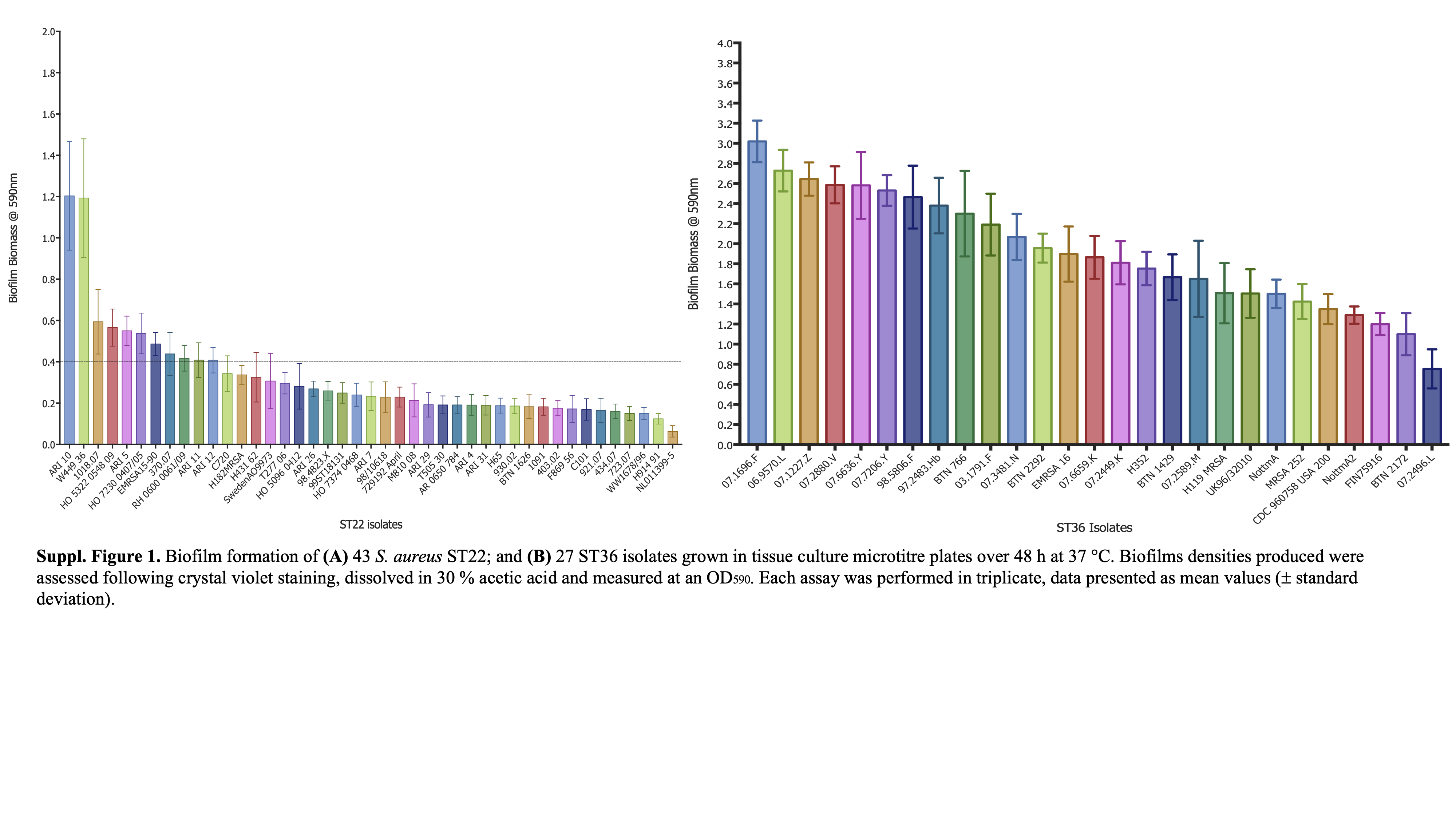

Supplement: Supplementary Figure 1 — Biofilm formation of (A) 43 S. aureus ST22; and (B) 27 ST36 isolates grown in tissue culture microtiter plates over 48 h at 37°C. Biofilms densities produced were assessed following crystal violet staining, dissolved in 30% acetic acid, and measured at an OD590. Each assay was performed in triplicate, data presented as mean values (± standard deviation). [file Image_1.jpeg]

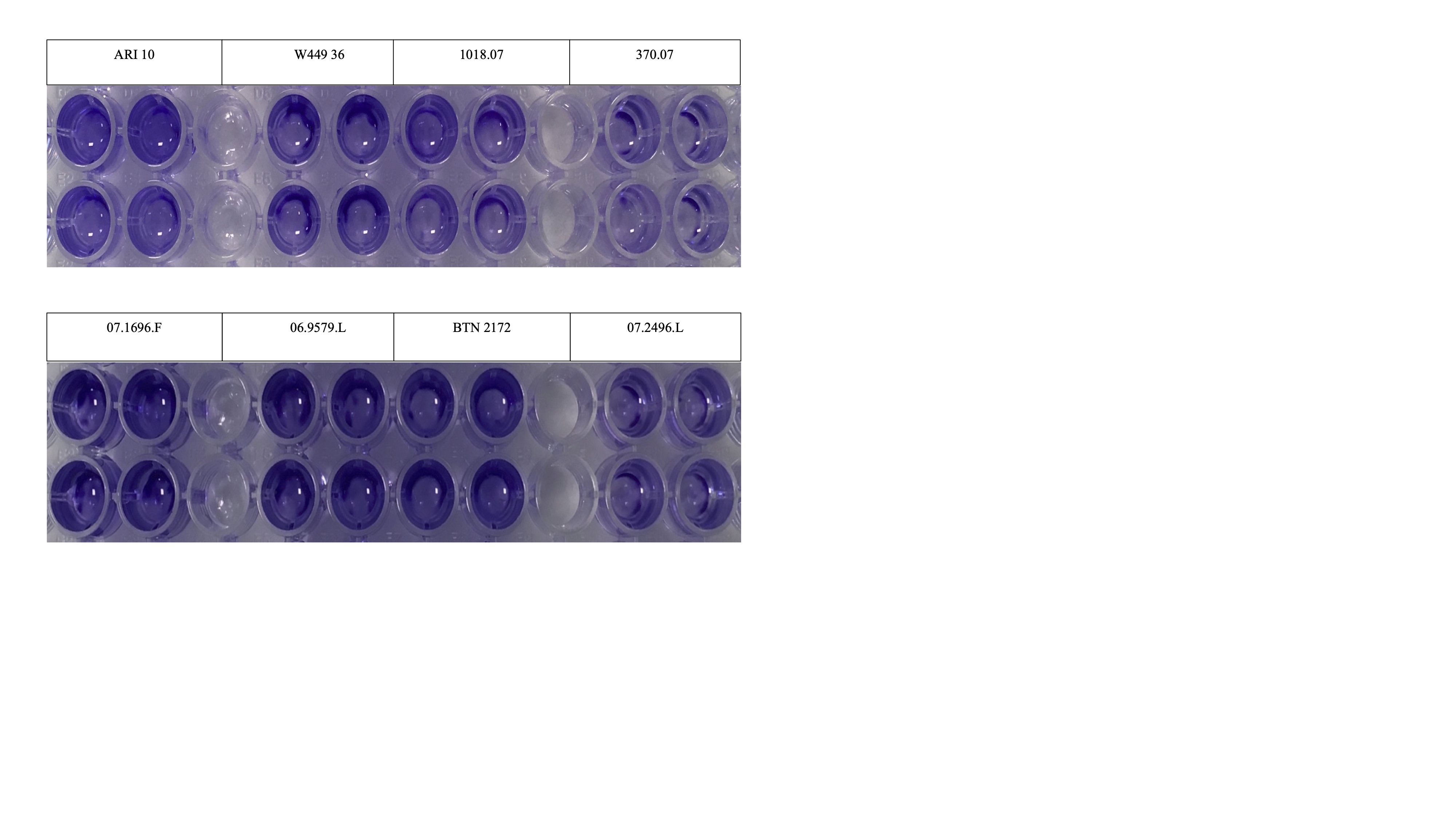

Supplement: Supplementary Figure 2 — Variation in biofilm production by ST22 (A) and ST36 (B) isolates after 48 h incubation in TSB supplemented with 1% D-(+)-glucose. Biofilms were visualized following staining with 0.1% crystal violet. [file Image_2.jpeg]

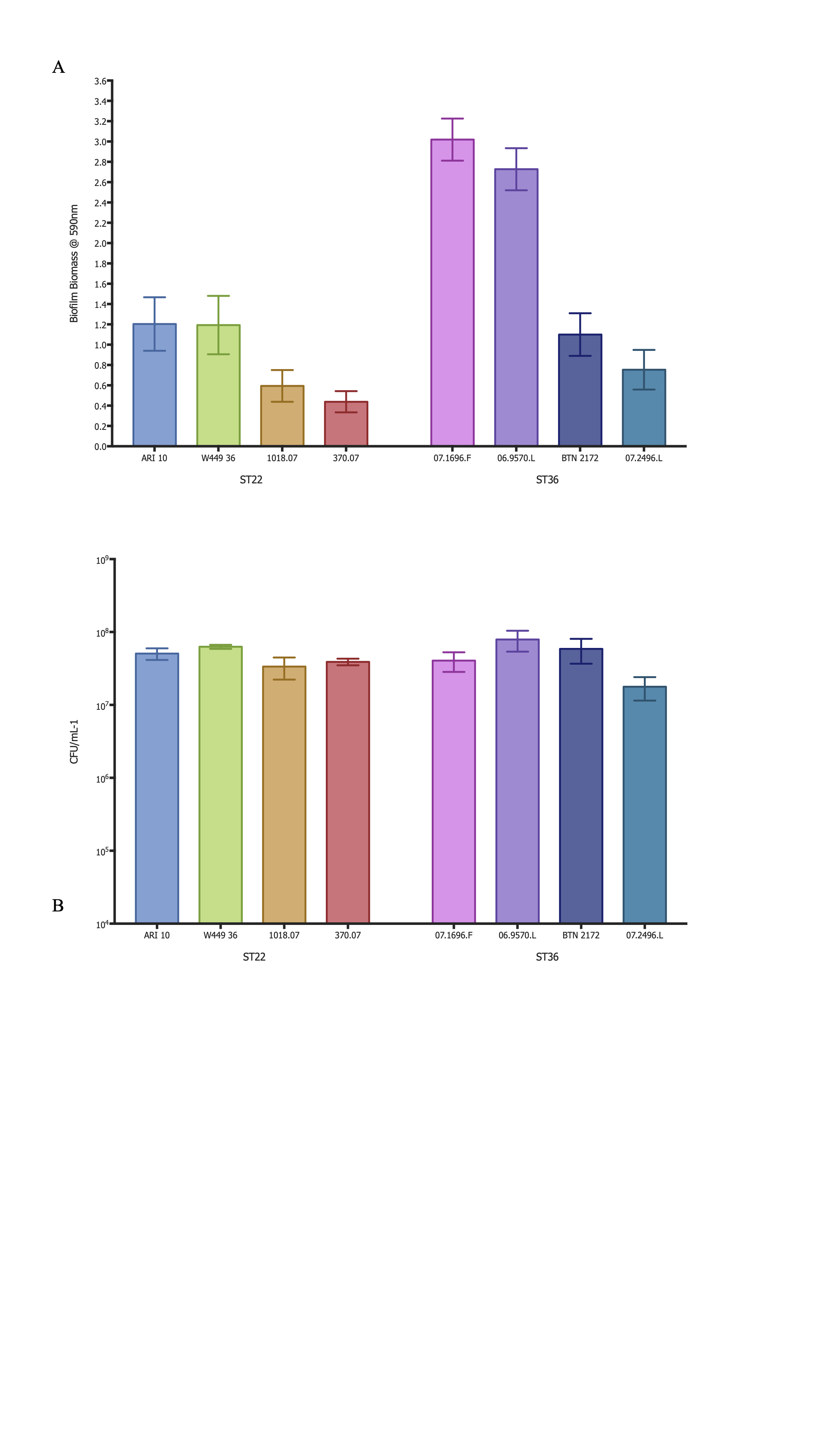

Supplement: Supplementary Figure 3 — Comparison of biofilm densities of eight S. aureus isolates assessed by CV staining (A) and corresponding viable cell counts (B). [file Image_3.jpeg]

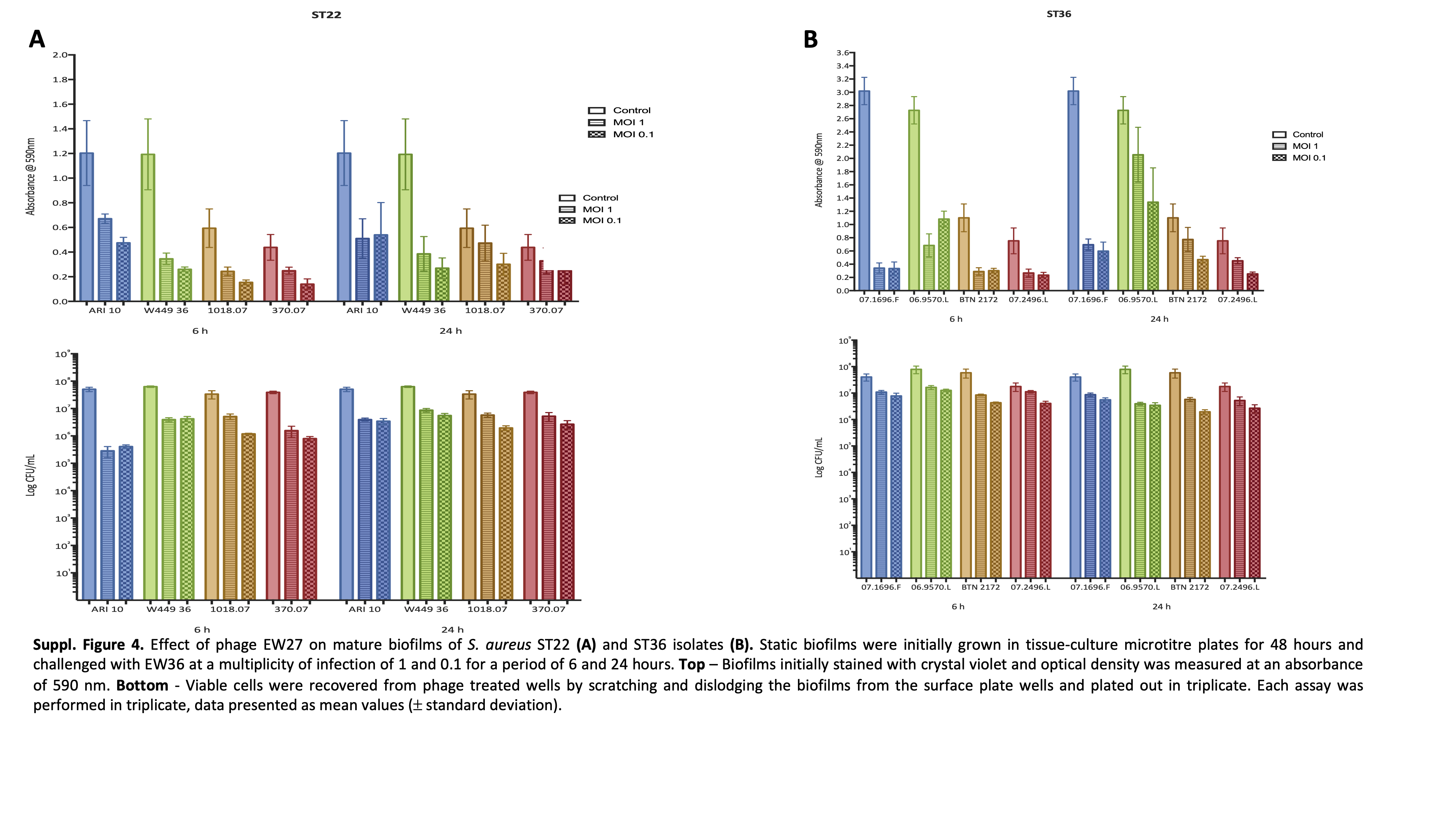

Supplement: Supplementary Figure 4 — Effect of phage EW27 on mature biofilms of S. aureus ST22 (A) and ST36 isolates (B). Static biofilms were initially grown in tissue-culture microtiter plates for 48 hand challenged with EW36 at a multiplicity of infection of 1 and 0.1 for a period of 6 and 24 h. (A) Biofilms initially stained with crystal violet and optical density was measured at an absorbance of 590 nm. (B) Viable cells were recovered from phage treated wells by scratching and dislodging the biofilms from the surface plate wells and plated out in triplicate. Each assay was performed in triplicate, data presented as mean values (± standard deviation). [file Image_4.jpeg]

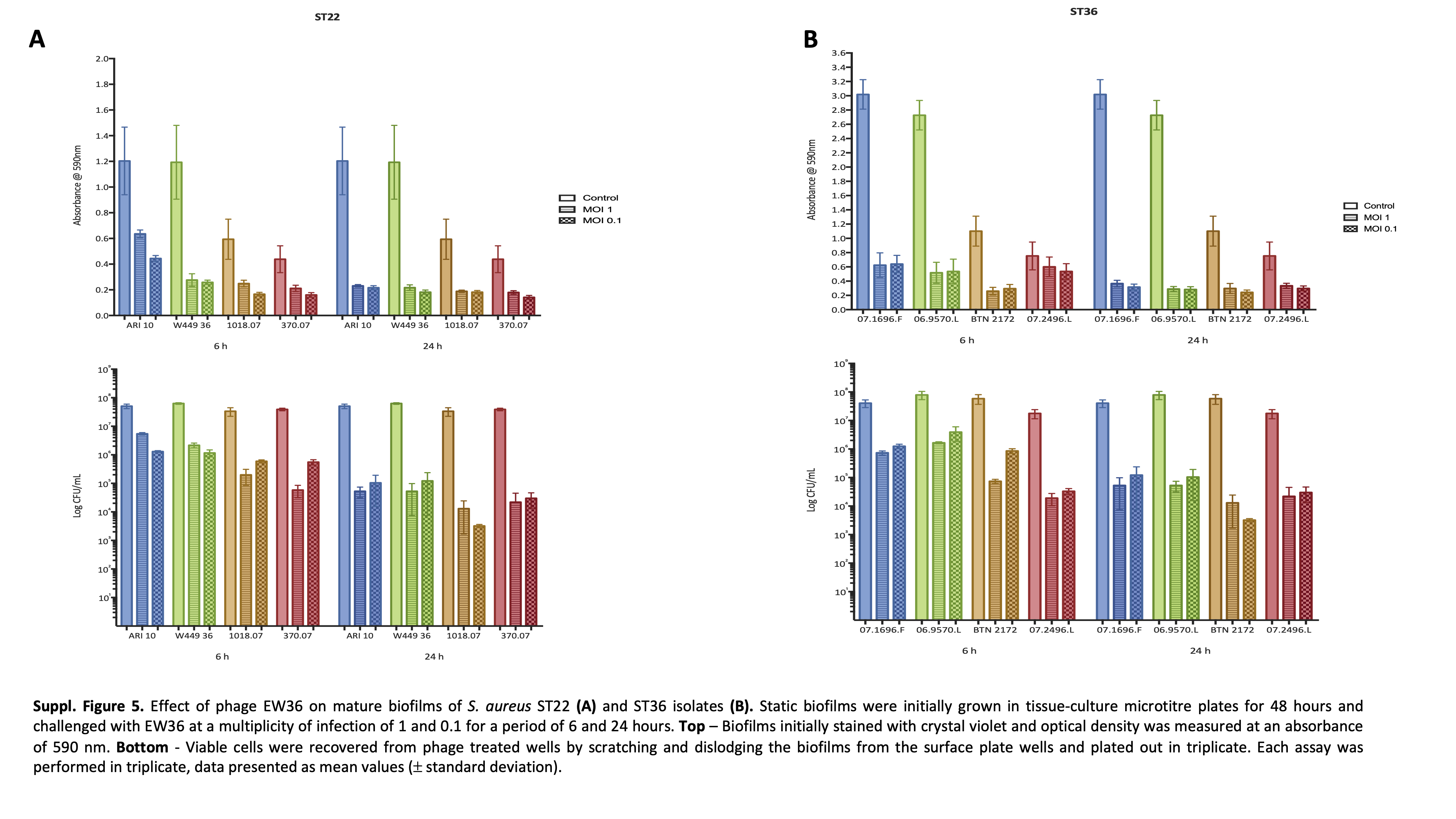

Supplement: Supplementary Figure 5 — Effect of phage EW36 on mature biofilms of S. aureus ST22 (A) and ST36 isolates (B). Static biofilms were initially grown in tissue-culture microtiter plates for 48 h and challenged with EW36 at a multiplicity of infection of 1 and 0.1 for a period of 6 and 24 h. Top Biofilms initially stained with crystal violet and optical density was measured at an absorbance of 590 nm. Bottom Viable cells were recovered from phage treated wells by scratching and dislodging the biofilms from the surface plate wells and plated out in triplicate. Each assay was performed in triplicate, data presented as mean values (± standard deviation). [file Image_5.jpeg]

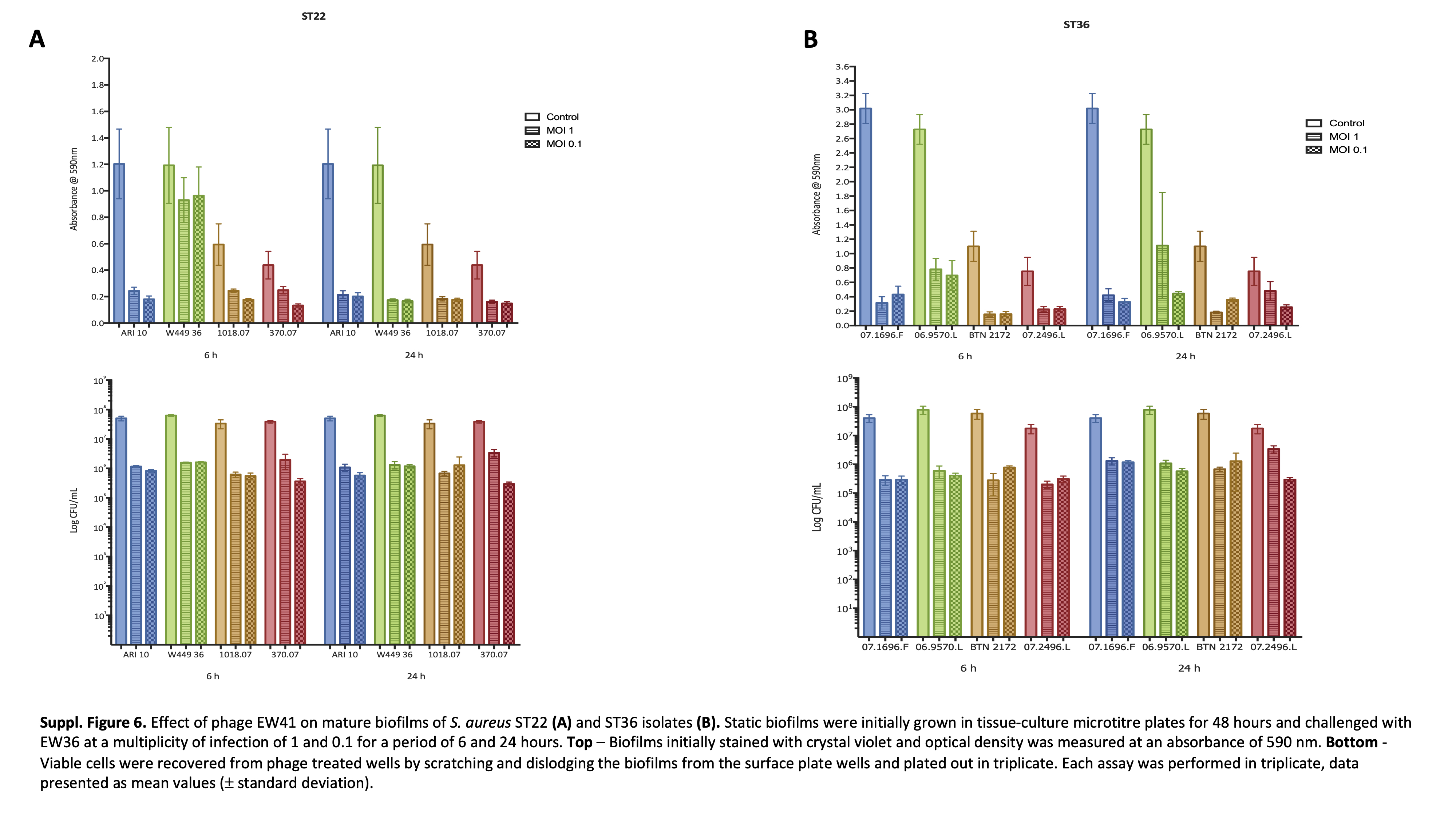

Supplement: Supplementary Figure 6 — Effect of phage EW41 on mature biofilms of S. aureus ST22 (A) and ST36 isolates (B). Static biofilms were initially grown in tissue-culture microtiter plates for 48 h and challenged with EW36 at a multiplicity of infection of 1 and 0.1 for a period of 6 and 24 h. Top Biofilms initially stained with crystal violet and optical density was measured at an absorbance of 590 nm. Bottom Viable cells were recovered from phage treated wells by scratching and dislodging the biofilms from the surface plate wells and plated out in triplicate. Each assay was performed in triplicate, data presented as mean values (± standard deviation). [file Image_6.jpeg]

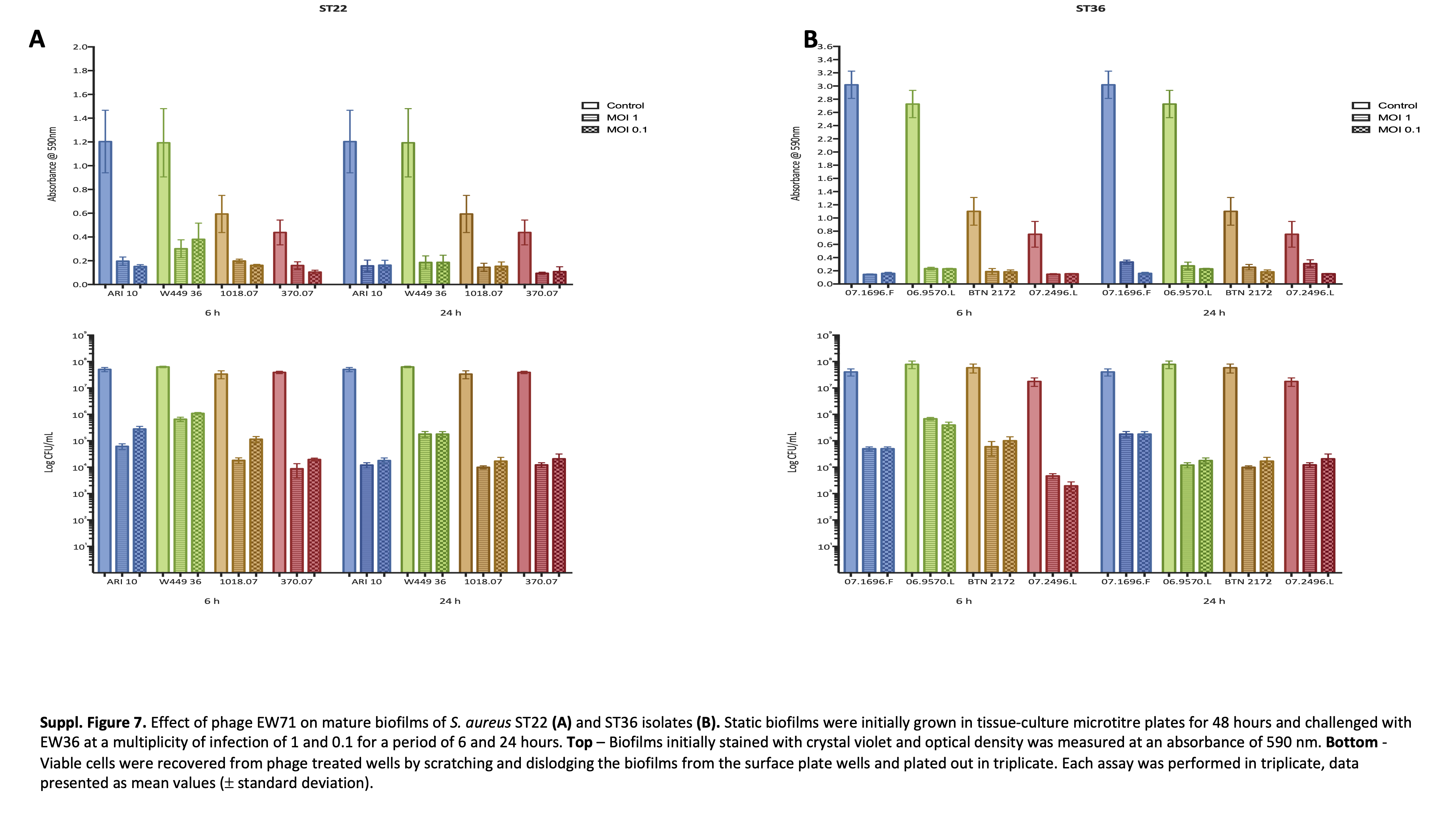

Supplement: Supplementary Figure 7 — Effect of phage EW71 on mature biofilms of S. aureus ST22 (A) and ST36 isolates (B). Static biofilms were initially grown in tissue-culture microtiter plates for 48 h and challenged with EW36 at a multiplicity of infection of 1 and 0.1 for a period of 6 and 24 h. Top Biofilms initially stained with crystal violet and optical density was measured at an absorbance of 590 nm. Bottom Viable cells were recovered from phage treated wells by scratching and dislodging the biofilms from the surface plate wells and plated out in triplicate. Each assay was performed in triplicate, data presented as mean values (± standard deviation). [file Image_7.jpeg]
